# Computing Critical Indices and Critical Thresholds of Effective Conductivity in Polydisperse Random Composites

**DOI:** 10.3390/ma19214492

**Published:** 2026-10-25

**Authors:** Vladimir Mityushev, Simon Gluzman, Joanna Kolodziej, Zhanat Zhunussova, Akylkerey Sarvarov

**Affiliations:** 1 Faculty of Computer Science and Mathematics, Krakow University of Technology, Warszawska St., 24, 31-155 Krakow, Poland; joanna.kolodziej@nask.pl; 2 Materialica + Research Group, Bathurst St. 3000, Apt. 606, Toronto, ON M6B 3B4, Canada; simongluzmannew@gmail.com; 3 Naukowa i Akademicka Siec Komputerowa-Panstwowy Instytut Badawczy (NASK-PIB), ul. Kolska 12, 01-045 Warszawa, Poland; 4 School of Applied Mathematics, Kazakh-British Technical University, Tole Bi Street 59, Almaty 050000, Kazakhstan; 5 Institute of Mathematics and Mathematical Modeling, Pushkina St., 125, Almaty 050010, Kazakhstan; akyl.sarvarov@gmail.com; 6 Faculty of Computer Technology and Cybersecurity, International Information Technology University, Manasa St., 34/1, Almaty 050040, Kazakhstan; 7 Faculty of Mathematics, Physics and Informatics, Abai Kazakh National Pedagogical University, Tole Bi Street 86, Almaty 050000, Kazakhstan

**Keywords:** densely packed composite, effective properties, network method, structural approximation, polydisperse composite, 74Q15

## Abstract

This paper is devoted to reviewing constructive methods for computing the effective conductivity (permeability and permittivity) of random, densely packed dispersed composites. The review is based on the formulation of boundary value problems within the framework of homogenization theory. The resulting problems can be solved using both numerical methods and analytical techniques realized as asymptotic methods. Particular attention is given to *a*RVE (analytical Representative Volume Element) theory based on the efficient evaluation of structural sums that characterize the geometric arrangement of inclusions, as these quantities play a crucial role in determining the critical parameters governing the effective properties of random dispersed composites. The resulting analytical expressions for the effective conductivity components $\lambda_{ij}^{\left(e\right)}$ can be obtained as polynomial functions of the inclusion concentration *f* valid from the dilute regime f=0 up to the critical concentration $f_{cp}$ corresponding to touching inclusions, or to the transport percolation threshold $f_{c}$. As *f* approaches $f_{cp}$, the composite becomes densely packed, and percolation chains may form. For materials with high contrast in their physical properties, the bounded polynomial behavior of $\lambda_{ij}^{\left(e\right)}$, as $f\to f_{c}$, may transition to a singular one, a phenomenon first identified by J. B. Keller in 1963. Beginning in 2001, L. Berlyand and A.G. Kolpakov extended Keller’s result to random composites and developed a theory describing pairwise interactions of inclusions of various shapes. These asymptotic developments were eventually unified into what is now known as the Network Method within the broader framework of Structural Approximation (SA). To maintain consistency with homogenization principles, we extend the SA framework by formulating a periodic boundary value problem on the corresponding Delaunay graph. This modification enables the application of SA to macroscopically anisotropic conducting composites. Special attention is given to elliptic inclusions governed by different probabilistic distributions. The analytical formulas obtained in this setting are then asymptotically transformed via renormalization techniques into expressions applicable to densely packed systems of ellipses. The method is extended to polydisperse composites containing circular inclusions of varying sizes.

## 1. Introduction

Densely packed composites are materials consisting of tightly arranged inclusions (particles, fibers, or grains) embedded within a matrix. These systems are common in advanced engineering materials such as ceramic composites [1,2] and particulate-reinforced polymers [3,4]. Their mechanical, thermal, and transport properties strongly depend on the spatial arrangement, volume fraction, and interaction of the constituent phases [5,6,7]. Mathematical models of densely packed composites integrate geometry, physics, and computation to link microscopic structure with macroscopic behavior [8]. Techniques such as the theory of homogenization discussed in seminal books [9,10,11], recent books [12,13], and percolation modeling [14] provide analytical insight, while numerical methods enable detailed local predictions [15,16]. Together, these approaches form a powerful framework for designing high-performance composite materials [17]. Such models capture force transmission in granular materials and load-bearing structures in packed composites. Experimental and numerical study of the thermal properties distribution in composite polycrystalline samples can be found in [18] and references therein. Analogous homogenization problems and investigation methods arise in biological structures [19] and the collective behavior of bacteria [20,21].

At high densities, perfectly conducting inclusions may form connected clusters, leading to percolation phenomena. This is crucial for thermal and electrical conductivity, fluid flow, and mechanical rigidity. A threshold $f_{c}$ is the critical concentration *f* above which a continuous path spans the material. The effective conductivity $\lambda_{e}$ (tensor or its components) of a composite with perfectly conducting inclusions has a singularity at $f=f_{c}$, asymptotically described by the following formula from different points of view: as development of crystalline [22], lattice and continuum percolation [14,23], and Padé approximation and renormalization [5], leading to the James Bond paradigm “shaken, not stirred” [5,24]

(1) $$\lambda_{e}≃{\left(f_{c}-f\right)}^{-s},\;as\;f\to f_{c},$$

where *s* is a critical exponent. One of the main problems of densely packed composites is the determination of the critical parameters $f_{c}$ and *s*. The computational experiments to determine $f_{c}$ were performed in [25,26] using the concept of a random jammed state. The comprehensive results on the determination of *s* are summarized in [24].

Throughout this work, we distinguish between two critical concentrations. The contact-percolation threshold $f_{cp}$ is defined as the volume fraction at which a system-spanning cluster of touching inclusions first emerges. In contrast, the transport threshold $f_{c}$ denotes the critical point at which the macroscopic effective conductivity loses analyticity and diverges in most cases. Near this point, the effective conductivity follows the critical scaling law (1). Neither $f_{c}$ nor *s* is universal; both depend sensitively on the microstructural architecture and, in particular, on the protocol used to generate the composite [24].

### 1.1. Percolation Effect

Classical percolation theory deals with lattice (site and bond) percolation [27], which describes a phase transition [28] and with continuum percolation of overlapping inclusions. This theory can be applied to densely packed composites within the framework of homogenization theory, as illustrated by the following example in the plane $\left(x_{1},x_{2}\right)\in R^{2}$. Let *Q* be the unit square representing a doubly periodic composite. Suppose that *N* perfectly conducting disks of radius *r* are embedded in an insulating host material. Overlap between disks is permitted. If a subset of the disks forms an overlapping chain connecting a pair of opposite sides of *Q*, we say that percolation occurs in the corresponding direction, for example, along the $x_{1}$-axis. Assume that the disk centers are fixed while the radius *r* increases from zero to a critical value $r_{c}$ at which percolation first occurs. This process corresponds to increasing the inclusion volume fraction *f* from zero to the critical value $f_{c}$ defined by Equation (1). Instead of disks, one can consider segments similar to Buffon’s needle, for which the fraction of inclusions *f* is considered to be the number of fractures per unit area [29].

The effective conductivity of the composite in the $x_{1}$-direction is zero for $f<f_{c}$ and diverges to infinity for $f>f_{c}$. Random configurations of overlapping disks can be generated using an homogeneous Poisson point process [30,31]. In this model, the coordinates of the disk centers are independent uniformly distributed random variables, so that each point has an equal probability of occurring anywhere within the unit square *Q*. In classical percolation theory, the two-point correlation (autocorrelation) function is often sufficient for studying critical parameters. The central problem of percolation theory is to determine the critical concentration $f_{c}$ and the critical exponent *s* for various overlapping inclusion geometries.

In the homogenization theory of densely packed composites with non-overlapping inclusions, the percolation problem is largely reduced to the same task of determining $f_{c}$ and *s*. One can find a theoretical attempt to describe the transition from the above 0-1 percolation conception to densely packed composites in [32]. The computational approach devoted to porous media was developed by P.M. Adler et al. [33] concerning the computational and theoretical approach to percolation and permeability of fracture networks [34,35].

One of the major computational challenges is the development of efficient formulas and algorithms for evaluating the multi-point correlation functions required for the determination of effective properties of random heterogeneous materials [36], since using only the two-point correlation functions reduces the study of the effective constants to $O(f^{3})$, as f→0. Moreover, the pure geometrical problem to estimate $f_{c}$ remains a demanding simulation problem [37]. Computational studies aimed at determining $f_{c}$ were carried out in [25,26] using the concept of the random jammed state. Comprehensive results concerning the determination of the critical exponent *s* are summarized in [24].

Further historical notes and aspects of rich percolation theory can be found in the above literature; see also [38,39,40] and references therein.

### 1.2. Pure Numerical Methods

We now consider random non-overlapping inclusions within the framework of continuum mechanics, where perfectly conducting inclusions are embedded in a matrix with normalized unit conductivity. Let $\lambda_{e}\left(f\right)$ denote the effective conductivity for volume fractions $f\leq f_{c}$ or $f\leq f_{cp}$. The function $\lambda_{e}\left(f\right)$ is assumed to increase monotonically with *f*, satisfying $\lambda_{e}\left(0\right)=1$, while the limiting values $\lambda_{e}\left(f_{cp}\right)$ and $\lambda_{e}\left(f_{c}\right)$ are finite and unbounded, respectively.

Within this framework, three representative scenarios can be distinguished. In the first scenario, the inclusion centers remain fixed while all inclusion sizes increase uniformly. At the critical volume fraction, neighboring inclusions may come into contact; however, a percolating chain is not necessarily formed, and therefore $\lambda_{e}\left(f_{cp}\right)$ can remain finite. In the second scenario, the inclusion sizes increase independently, allowing a connected chain of touching inclusions to form at $f=f_{c}$. In this case, the effective conductivity may diverge. The third scenario, in which the inclusion locations are random, essentially differs from the described deterministic case above and constitutes the theory of random dispersed composites beginning from [41,42]. The conception of random structures suitable for high concentrations will be discussed in Section 1.4.

Finite Element Methods (FEM) are powerful computational tools for solving boundary value problems in domains with prescribed geometries and material properties [43,44]. They enable detailed analyses of local fields, stress distributions, and transport phenomena [45,46]. Such analyses are well suited to the first scenario and, under appropriate conditions, may also be applied to the second. A comprehensive discussion of the strengths and limitations of FEM is given in [47].

The third scenario, involving random dense composites, presents substantially greater computational challenges than those typically implied by commercial FEM software. Reliable stochastic characterization requires a sufficiently large number of Monte Carlo realizations to capture the variability of the microstructure, often resulting in prohibitively high computational costs. Moreover, the direct application of FEM to densely packed composites is hindered by the presence of extremely narrow gaps between neighboring inclusions. Accurate resolution of these regions requires highly refined meshes because the local fields exhibit singular or near-singular behavior. As a consequence, the computational cost increases drastically, making conventional finite element analyses inefficient or even impractical for large-scale stochastic investigations of dense composite materials.

As noted above, it is difficult to investigate a singular behavior by finite elements, which are adopted to bounded local fields a priori. This study concerns all types of methods based on equations with a bounded operator in an appropriate space; see, for instance, ref. [48], where an increasing number of iterations was explicitly constructed to determine the local field between two approaching inclusions. The exact solution to the two-disk problem was solved in [49]. Refs. [15,16] deserve special attention, where a comprehensive FEM analysis was performed to show the local stress–strain state in the reinforced plate with orthogonal systems of fibers.

The incorporation of asymptotic formulas can effectively address the difficulties caused by singular fields arising in narrow gaps between highly conducting inclusions [50,51] and in the vicinity of crack tips [52,53]. In fracture mechanics, modern FEM packages routinely employ asymptotic enrichment techniques, whereby the singular component of the solution, typically expressed through stress-intensity factor-dependent terms, is separated from the regular component computed numerically. This strategy significantly enhances both accuracy and computational efficiency. To the best of our knowledge, however, analogous asymptotic enrichment methods for the singular fields that develop between closely spaced highly conducting inclusions have not yet been incorporated into standard commercial FEM packages.

The method of integral equations belongs to the class of numerical methods. In homogenization theory, boundary value problems are considered in a periodic setting or within bounded domains possessing certain symmetry properties. These formulations require integral representations of local fields involving periodic kernels. Two-dimensional periodic conductivity and elasticity problems were systematically investigated in [54,55] by means of complex potentials. Effective numerical algorithms were developed in [56,57] for 2D composites. A method for 3D composites was developed in [58]. The classic methods of non-periodic integral equations were developed in [59]. These methods have been successfully applied to the simulation of local fields and can also be employed for homogenization analysis [60]. A major advantage of integral equation methods is that they are formulated on the boundaries of inclusions, allowing them to capture local field behavior with high accuracy. Nevertheless, the numerical treatment of composites containing multiple inclusions with closely spaced or touching closed boundaries remains challenging for applications to densely packed microstructures.

### 1.3. Empirical Analytical Approaches

One of the most popular formulas, Rule of Mixtures “has been overused and applied to cases that do not even come close to respecting its original, simplifying assumptions” [61]; see the extensive analysis in [62]. This formula is justified for laminated composites by solution to the corresponding 1D homogenization problem. However, other empirical formulas and methods, such as the Mori–Tanaka scheme, fail to provide accurate predictions for densely packed composites.

Reviews of various empirical analytical formulas for estimating the effective properties of composites often begin by presenting a broad collection of methods and their numerous modifications. Yet, many of these approaches are inconsistent with one another; some contradict established theoretical bounds [63,64], while others fail to preserve tensor symmetries. As a result, theoretical rigor is often replaced by empirical reasoning, with partial experimental agreement serving as the primary justification for introducing new models.

On the one hand, empirical formulas are frequently used due to the strong trend of their universality, in particular, weak dependence on geometry; see, for instance, [65] and the recent papers [66,67]. At the same time, there is a permanently expanding set of various empirical formulas and over-simplified numerical procedures for effective constants. As an example, consider the special empirical model for densely packed polymer composites proposed in [68], where one-dimensional empirical approximations were used for the effective conductivity of body-centered cubic structure. The author confirmed excellent agreement with experimental data, though a singular percolation behavior similar to [69] was not observed.

A comparison between the three-dimensional effective elastic constants computed by FEM and those predicted by a cluster interaction model for cells containing 50 spherical inclusions was presented in [70]. In that study, both the inclusion locations and the elastic constants of the two-phase composite were fixed numerically. The cluster interaction model, also referred to in the literature as the effective medium approximation or the self-consistent approach (see the references cited in [70]), is formulated for effective properties averaged over a finite domain and therefore does not satisfy the classical homogenization principles discussed in [71]. Consequently, the resulting expressions are generally valid only for dilute composites, as demonstrated for two-dimensional thermoelastic composites in [72]. More specifically, they provide correct asymptotic expansions up to the second, and in some cases third order, in the inclusion volume fraction *f*, as f→0. From this perspective, the discrepancy between the approaches reported in [70] is expected, since they effectively describe two different classes of composites. The observed numerical differences, however, may remain relatively small. This can be explained by asymptotic estimates involving the dimensionless contrast parameters $\varrho_{1}=0.6$, $\varrho_{2}=0.7$ and $\varrho_{3}=0.5$ introduced in [73]. According to the analytical asymptotic formulas derived in [73], the discrepancy is of order $\varrho_{2}\varrho_{3}f$, which yields approximately 0.07 to 0.2 for the numerical examples considered in [70]. This estimate also indicates that the empirical packing parameter $\frac{d}{2r}$, where *d* denotes the mean nearest-neighbor distance between inclusions and *r* their radius, should not be regarded as a fundamental descriptor of densely packed composites. Instead, the relevant packing parameters arise naturally from asymptotic analysis, as discussed in the next section. In particular, this framework leads to the key parameters $f_{c}$ and *s* defined in Equation (1), which govern the behavior of densely packed composites.

### 1.4. Asymptotic Analytical Method (AAM)

Asymptotic Analytical Methods (AAMs) combine geometric modeling, physical descriptions, and rigorous analytical techniques to overcome the limitations of the numerical and empirical approaches discussed in Section 1.2 and Section 1.3. A key feature of AAMs is their ability to quantify approximation errors analytically through the Big *O* symbol, commonly referred to as the Bachmann–Landau notation. In particular, asymptotic methods provide explicit formulas for effective conductivity together with rigorous error estimates, for example, up to $O(f^{2})$ as the inclusion concentration f→0, or up to O(δ) as the gap between neighboring inclusions δ→0. This capability represents a significant advantage over empirical and purely numerical methods. Unlike empirical formulas, asymptotic results are accompanied by well-defined accuracy bounds; unlike numerical simulations, they reveal the underlying singular behavior of the physical system and elucidate the dependence of the solution on material and geometric parameters. Consequently, asymptotic analytical methods play a central role in the study of densely packed composites, where complex particle configurations and strong inter-particle interactions often render purely numerical approaches computationally demanding.

The conception of random structures considers geometries as random fields governed by probability measures rather than as deterministic constants [9,74]. Dispersed random densely packed composites are covered by the general theory based on multi-point correlation functions proposed in [41] and developed in [36]. However, it is not acceptable for higher concentrations, since *n*-point correlation functions produce polynomial approximations of degree *n* in the concentration and contrast parameter [5,36]. Computation of *n*-point correlation functions for n≥4 is virtually impossible, which does not allow us to obtain the higher terms needed for densely packed composites.

The necessary formulas can be obtained by different alternative ways described beginning from Section 3 in the following way. First, analytical exact and approximate asymptotic formulas and symbolic computational algorithms are considered formally in the deterministic setting. The geometric parameters such as the gravitational centers of inclusions $A=\{a_{k}\in Q,\;k=1,2,\ldots ,N\}$, static moments of the inclusion’s shape and others are considered symbolically [75]. At the final step, when a formula for the effective constant $\lambda_{e}$ is derived, the geometric parameters are considered as random variables and the effective constant of random composites is determined, for instance, as the expectation $\langle \lambda_{e}\left(A\right)\rangle$ over all the considered probabilistic events. Numerically, the expectation can be calculated by Monte Carlo simulations [5].

The present review is devoted to the accurate modeling of densely packed microstructures by AAMs and to the development of a detailed strategy for computing the effective conductivity of dispersed composites with densely packed random inclusions along the lines described above. The computational generation of inclusions was described in [24] and the work cited therein. They include different protocols based on the Random Sequential Addition (RSA), when particles are placed sequentially without overlap. Various modifications of RSA were developed and statistically investigated. It was demonstrated that the critical index *s* should not be regarded as universal but should be seen as a spectral response to the continuous breaking of translational symmetry.

In the present paper, we extend the approach proposed in [24] to polydisperse systems. In addition to outlining the general framework, we develop a symbolic-numerical algorithm applicable to various inclusion shapes. Particular attention is devoted to elliptical inclusions, for which we derive new approximate analytical formulas. In addition, the formulas previously developed for random conducting composites containing non-overlapping elliptical inclusions emerge as particular cases of the more general expressions derived in the present paper. The theoretical justification follows from the precision of approximations already established in [76] through the application of Schwarz’s alternating method combined with asymptotic analysis for inclusions of arbitrary shape. The obtained results provide deeper insights into the relationship between microstructural morphology and the effective properties of composite materials.

The results are presented in the form of steps which should be performed to estimate the effective conductivity of composites with highly conducting inclusions. In Section 3, we outline network approximations and the method of structural approximations [77,78,79] summarized in the books [80,81] and the paper [82], which provides the leading term of $\lambda_{e}$ near the percolation point $f=f_{c}$. It is worth noting that the method of structural approximations is based on local particle interactions based on the asymptotic relations similar to (1). In Section 4 and Section 5, we take into account the global approximations by constructing expansions of $\lambda_{e}=\lambda_{e}\left(f\right)$ at the point f=0. The developed method yields analytical formulas for $\lambda_{e}$ and an algorithm based on the Padé approximants and the resummation method to determine the critical global parameters $f_{c}$ and *s*. To summarize, we develop a strategy for investigating densely packed composites shortly based on the following steps:

- Local study of the interparticle interaction.
- Construction of the Voronoi tessellation and Delaunay triangulation (graph) to apply the structural approximation to numerically estimate the effective conductivity.

The above scheme can be enriched by the method based on the *a*RVE theory [5,75]

- Expansions $\lambda_{e}$ at the point f=0.
- Computation of the critical parameters $f_{c}$ and *s*.

While generalized transport theories encompass a vast array of continuum models and complex multi-phase media, the present study restricts its scope strictly to two-dimensional, two-phase, multi-scale random composites near high packing limits. Our primary objective is not to survey arbitrary heterogeneous media, but rather to investigate how localized microstructural fluctuations within specific stochastic realizations govern the convergence of structural sums. By strictly confining our analysis to this well-defined class of densely packed dispersions, we establish a precise methodological framework for evaluating sample-to-sample variations in critical thresholds $f_{c}$ and transport exponents *s*.

The structural approximations were developed for concentrated suspensions in viscous fluids [83]. However, despite the recent success in description of local elastic fields [84,85], the network method is not sufficiently well developed yet for densely packed composites. An AAM was applied to 3D conductivity and to 2D elastic and viscous media [73].

The central theme of this review is to construct a comprehensive, unified framework for evaluating the effective macroscopic transport properties of heterogeneous composites, particularly focusing on the critical behavior near the phase transition. To guide the reader through the diverse methodologies required to address this problem, the remainder of the manuscript is organized as follows. In the present Section 1, we establish the fundamental physical principles of percolation, emphasizing continuous models, threshold concentrations $f_{c}$, and protocol-dependent critical indices. We evaluate existing empirical models, assessing their practical utility and underlying physical limitations in capturing singular behavior. To systematically move beyond empirical approximations, we describe in Section 2 the rigorous problem formulation necessary to simulate macroscopic transport in structurally complex media. Section 3 details the structural approximation and network approximation, which provides a vital mechanistic bridge by modeling the gap conductances between closely packed inclusions. The final sections address specific geometric complexities inherent in realistic structured media: Section 4 analyses the distinct mathematical and physical challenges posed by elliptic inclusions. Section 5 extends our theoretical framework to polydisperse composites. Section 6 introduces asymptotic analytical methods, demonstrating how advanced approximation techniques can extract physical parameters from divergent perturbation series; illustrating how variance in inclusion size distributions dictates the onset of the percolation threshold and effective critical indices. We conclude in Section 7 by summarizing our main findings and outlining future directions for theoretical and computational research.

## 2. Structural Approximation: Periodic Boundary Value Problem

First, we state a periodic boundary value problem for the conductivity of a composite cell according to the homogenization theory, which extends the consideration of composites by studying them in a finite domain [86]. For definiteness, a two-dimensional problem with circular inclusions in the unit square cell is considered. Elasticity and three-dimensional problems are stated analogously [9].

Introduce the lattice $Q=\{k_{1}\omega_{1}+k_{2}\omega_{2}:\;k_{1},k_{2}\in Z\}$ generated by two fundamental translation vectors $\omega_{1}=\left(1,0\right)$ and $\omega_{2}=\left(0,1\right)$ on the plane. The fundamental domain of the plane torus corresponding to the lattice Q is the unit square

(2) $$Q=\left\{\left(x_{1},x_{2}\right):-\frac{1}{2}<x_{1,2}<\frac{1}{2}\right\}.$$

**Figure 1:**
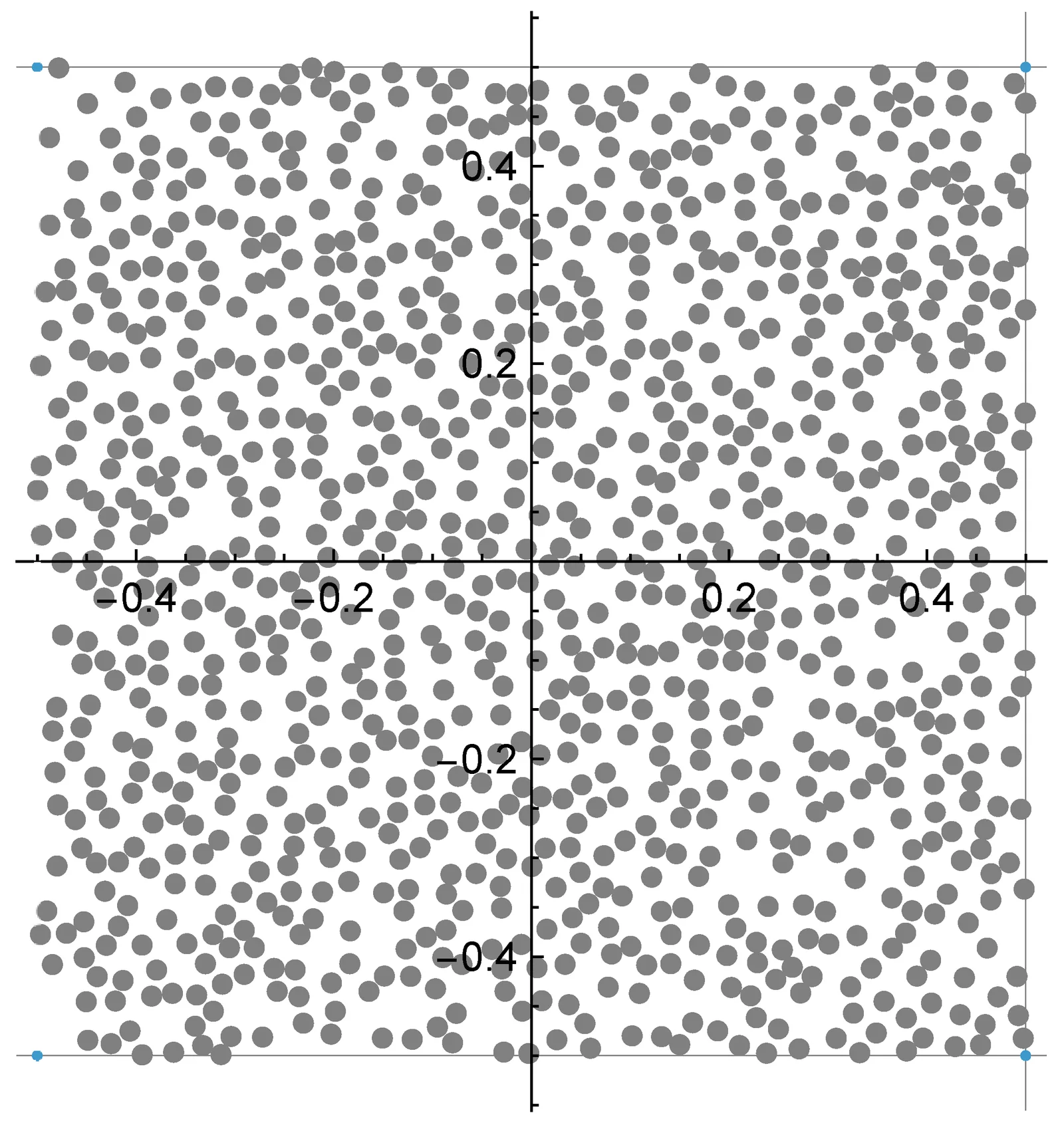
The fundamental unit square cell *Q* given by (2) with N=1000 inclusions per cell.

Consider non-overlapping inclusions $D_{k}$ (k=1,2,…,N) in the considered torus topology. Introduce the domain *D*, the complement of the closure of $D^{+}=\cup_{k=1}^{N}D_{k}$ to *Q*. Figure 1 displays an example of the structure considered for the non-overlapping disks $D_{k}=\{x\equiv \left(x_{1},x_{2}\right):|x-a_{k}\left|<r_{k}\right\}$ (k=1,2,…,N) mainly used throughout the paper.

Let *D* be occupied by a material of normalized unit cell conductivity and the inclusions $D_{k}$ be perfect conductors having infinite conductivity. The function u(x) is harmonic in *D* and continuously differentiable in its closure. It satisfies the boundary conditions [80,81]

(3) $$u\left(x\right)=u_{k},\;x\in \partial D_{k},\;\int_{\partial D_{k}}\frac{\partial u}{\partial n}\left(x\right)dx=0,\;k=1,2,\ldots ,N,$$

where the undetermined constants $u_{k}$ can be found during the solution to the problem.

It is assumed that u(x) satisfies the jump conditions

(4) $$u\left(x_{1}+1,x_{2}\right)-u\left(x_{1},x_{2}\right)=1,\;u\left(x_{1},x_{2}+1\right)-u\left(x_{1},x_{2}\right)=0.$$

The conditions (4) imply that the averaged gradient 〈∇u〉 over *Q* is equal to the unit vector ${\left(1,0\right)}^{T}$. The effective conductivity tensor $\lambda_{e}$ can be calculated by the following formula [5] (Section 2.2.2) and [9]:

(5) $$\langle \lambda \left(x\right)\nabla u\rangle =\lambda_{e}{\left(1,0\right)}^{T}.$$

It is worth noting that the problem (3) and (4) can be considered in Sobolev spaces following [9,81].

## 3. Structural Approximation and Network Model

Dense composites with a high inclusion concentration exhibit strong interactions between neighboring inclusions, dominated by the singular behavior of the fields in narrow gaps. Structural Approximation provides a systematic way to reduce the full continuum problem to a network problem by isolating and characterizing these singular interactions. The resulting network captures the essential geometry of the composite near the touching concentration $f_{c}$. Section 3.1 summarizes the main formulas for the interparticle flux interaction (capacity) between pairs of inclusions. Section 3.2 contains the constructive part of the structural approximation demonstrating how to apply the pair interactions to a general network of interacting particles.

### 3.1. Narrow Gaps and Singular Interactions

When two inclusions approach each other, the local field (electric, anti-plane elasticity, or thermal) becomes strongly concentrated in the narrow gap separating them. Let $\delta_{mk}=\inf_{x\in D_{m},y\in D_{k}}∥x-y∥$ denote the gap between the neighbor inclusions $D_{m}$ and $D_{k}$ in the toroidal double periodic metric. In the case of circular inclusions, we have $\delta_{mk}=∥a_{m}-a_{k}∥-r_{m}-r_{k}$, where in the considered metric $∥a_{m}-a_{k}∥:=\min_{l_{1},l_{2}=-1,0,1}\left|a_{m}-a_{k}+l_{1}+il_{2}\right|$ [87]. In 1963, J.B. Keller [69] considered a regular square array of ideally conducting circular inclusions of radii *r*, and estimated the interparticle flux interaction (capacity, flux per unit difference of potentials) through the gap distance

(6) $$g_{mk}=\pi \sqrt{\frac{r}{\delta }}+O\left(1\right),\;as\;\delta \to 0,$$

where the dimensionless gap $\delta =\frac{1-2r}{r}$ is used. The asymptotic (6) immediately yields the formulas for the effective conductivity of the square and hexagonal arrays (|Q|=1) of disks [5] (p. 74) and [69]

(7) $$\lambda_{sq}=\frac{\pi }{\sqrt{\delta }}+O\left(1\right),\;\lambda_{hex}=\frac{\sqrt{3}\pi }{\sqrt{\delta }}+O\left(1\right),\;as\;\delta \to 0,$$

where $\delta =\frac{\sqrt[{4}]{\frac{4}{3}}-2r}{r}$ holds for the hexagonal array. The effective conductivity of a regular cubic array of spherical inclusions was estimated by the flux interaction [69] $g_{mk}=-\pi r\ln\delta +O\left(1\right)$.

The seminal Keller’s Formula (6) was rigorously justified in [80,81] by means of a variational method. In addition, books [80,81] developed the following two fundamental steps for general densely packed composites:

1. A systematic study of the interparticle flux interaction between various shapes of inclusions.
2. Development of the network approximation to an arbitrary location of inclusions in *Q*, as shown in Figure 1. Computation of the singular part of the effective tensor, as $\delta_{max}=\max_{km}\left(\delta_{km}\right)\to 0$.

Following [80], we provide some examples of two-body interparticle flux (capacities) and general observations.

- The planar capacitor in $R^{3}$ of area *s* has the capacity $\frac{s}{\delta }$.
- The capacity of a cone and plane pair is bounded in $R^{3}$. This means that $g_{mk}=O\left(1\right)$, as $\delta_{km}\to 0$. The same estimation holds for a pair of polyhedrons in the general position.
- The capacity of two spheres of radii $r_{k}$ and $r_{m}$ in $R^{3}$ holds
  (8) $$g_{mk}=-2\pi \frac{r_{k}r_{m}}{r_{k}+r_{m}}\ln\delta_{km}+O\left(1\right),\;as\;\delta_{km}\to 0.$$
  We have $g_{mk}=-\pi r\ln\delta_{mk}+O\left(1\right)$ for equal radii.
- The capacity of a pair of four-power paraboloids $x_{3}=\pm \frac{1}{2}\left[{\left(x_{1}^{2}+x_{2}^{2}\right)}^{2}+\delta \right]$ holds $\frac{\pi }{\sqrt{\delta }}+O\left(1\right)$.
- The capacity of a pair of two different disks in $R^{2}$ equals
  (9) $$g_{mk}=\frac{\pi }{\sqrt{\delta_{km}}}\sqrt{\frac{2r_{k}r_{m}}{r_{k}+r_{m}}}+O\left(1\right).$$
- The lower bound $C\ln\frac{1}{\delta }$ on the angle-line capacity in $R^{2}$ is established. This is extended to the pair of polygons.

Following [88,89], we add some results concerning the nonlinear p−Laplacian equation $\nabla \cdot (|\nabla u{|}^{p-2}\nabla u)\;=0$ in *D* (p>2). The capacity of two spheres of radius *r* in $R^{3}$ is equal to

(10) $$g_{mk}=\frac{\pi }{\left(p-2\right)r^{p-3}}{\left(\frac{2}{\delta_{mk}}\right)}^{p-2}+O\left(1\right).$$

The capacity of two disks in $R^{2}$ is equal to

(11) $$g_{mk}=\frac{(2p-5)!!}{(2p-4)!!}\frac{\pi^{\frac{3}{2}}}{r^{p-2}}{\left(\frac{2}{\delta_{mk}}\right)}^{p-\frac{3}{2}}+O\left(1\right).$$

The capacity of two spheres in $R^{d}$ has a singular behavior only for $p>\frac{d+1}{2}$ [88,90,91]. For definiteness, write the formula for an even number *d*

(12) $$g_{mk}=\delta_{mk}^{-p+\frac{d+1}{2}}\frac{{\left(\pi r\right)}^{\frac{d-1}{2}}\left(p-\frac{d+3}{2}\right)!}{(p-2)!}+O\left(1\right).$$

In the case $p\leq \frac{d+1}{2}$, the capacity becomes bounded, particularly for the balls for Laplace’s equation (p=2) in $R^{d}$ for d≥4.

### 3.2. Delaunay Graph and Network Representation

The second fundamental achievement of the network approximation [80,81] is the constructive application to the effective conductivity of randomly densely packed composites. In 1927, I.E. Tamm [92] discussed the shielding (tunnel) effect of a system of bodies, where the capacity of two closely placed bodies does not depend on other bodies which are far away from these two bodies. The network approximation [80,81] may be considered as a development of Tamm’s physical discussion of the shielding effect and Keller’s asymptotic formula [69] from the point of view of asymptotic theory and homogenization.

The corresponding boundary value problem in continuous and discrete statements was used in [80,81]. In the present paper, the problem for a domain *Q* is modified to the periodic statement (3) and (4) following the principles of homogenization [71,82,93].

**Figure 2:**
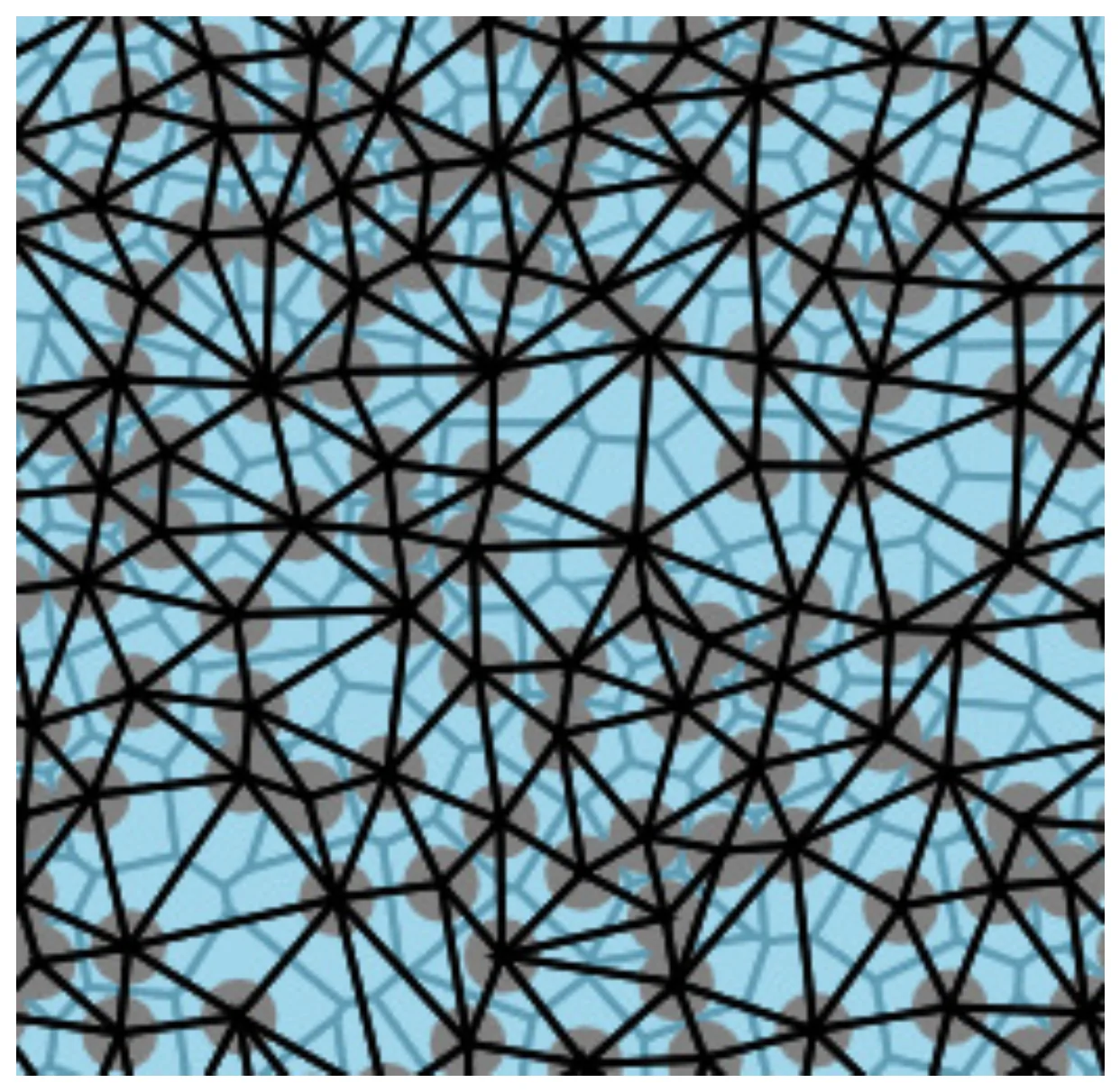
The Voronoi tessellation of the central fragment of Figure 1. The vertices of the Delaunay graph coinciding with the centers of Voronoi domains are the centers of inclusions.

The Voronoi diagram and Delaunay triangulation displayed in Figure 2 must be used on a torus as explained in [88] for the periodic statement of the problem. The centers of the inclusions define a Delaunay graph, whose edges connect nearest neighbors. Each edge corresponds to a potential narrow gap and is assigned a weight determined by the asymptotic interaction $g_{ij}$. The resulting weighted graph represents the transport or load-bearing backbone of the composite. The effective property of the composite is then approximated by the effective property of the corresponding network. For conductivity, this is the effective conductance of the network; for elasticity, it is the effective stiffness of the network under appropriate loading conditions. The network inherits the statistical properties of the underlying microstructure, including the effects of the randomness protocol.

The neighborhood relation between the two vertices is denoted by $a_{j}∼a_{k}$ or just j∼k for simplicity. The doubly periodic graph (V,E) connecting the centers of neighboring inclusions will be called the Delaunay graph. Here, *V* denotes the vertex set, and *E* is the edge set. For each fixed $a_{k}$, introduce the set of neighbor vertices $J_{k}=\cup_{j∼k}\;a_{j}$. Their total number $N_{k}=\#J_{k}$ is the degree of the vertex $a_{k}$. A number $u_{k}$ is assigned to each $a_{k}$ and $g_{mk}$ to each edge $e_{j}$ connecting $a_{k}$ and $a_{m}$. The flux arising in the network (V,E) satisfies the Kirchhoff balance equations:

(13) $$\sum_{j∼k}g_{kj}\left(u_{k}-u_{j}^{*}\right)=0,\;k=1,2,\ldots ,N,$$

where the sum $\sum_{j∼k}$ contains $N_{k}$ terms. Here, $u_{j}^{*}=u_{j}$ if the corresponding edge of $J_{k}$ connects $a_{k}$ with $a_{j}\in Q$ or with $a_{j}\pm \left(0,1\right)$. Otherwise, $u_{j}^{*}=u_{j}\pm 1$, i.e., the edge connects $a_{k}$ with $a_{j}\pm \left(1,0\right)$. This condition on $u_{j}^{*}$ determines the external flux (1,0). Equation (13), with an arbitrarily fixed $u_{N}$, has a unique solution.

Let a solution of (13) be found. Then, the average field energy stored in the composite is calculated as the sum of edge energies:

(14) $$E=\frac{1}{4}\sum^{\left(G\right)}g_{kj}{\left(u_{k}-u_{j}^{*}\right)}^{2},$$

where the notation $\sum^{\left(G\right)}=\sum_{k=1}^{N}\sum_{j∼k}$ is used for simplicity. The multiplier $\frac{1}{4}$ arises due to the double counting of edges. Equation (13) is obtained from the stationary points of (14), when $\frac{\partial E}{\partial u_{k}}=0$ (k=1,2,…,N).

The effective conductivity is determined by the macroscopic energy equation $E=\frac{1}{2}\lambda_{e}{\left[u_{0}\right]}_{1}^{2}$, where the increment ${\left[u_{0}\right]}_{1}$ of the macroscopic temperature $u_{0}\left(x\right)=x_{1}$ per unit length is normalized to unity. Therefore, the effective conductivity of densely packed macroscopically isotropic disks can be estimated from the formula $\lambda_{e}=2E\left(u\right)$.

We now turn to an extension of the network approximation introduced in Section 3.2 to the case of densely packed inclusions of arbitrary shape. As observed in [69] and rigorously justified in [80,81], the capacity of two smooth inclusions in two dimensions is captured by the asymptotic Formula (9). This relies on the fact that, for 2D curves, it is sufficient to perform local calculations up to third-order terms in the boundary parametrization leading to the same asymptotic formula for capacity. For instance, the parabola $y=x^{2}$ and the circle $x^{2}+{\left(y-\frac{1}{2}\right)}^{2}=\frac{1}{4}$ have the same curvature radius $\frac{1}{2}$ at x=0; therefore, each forms with the line y=−δ a pair of identical capacity up to O(1), as δ→0. An analogous statement, however, no longer holds for surfaces in $R^{3}$, where the geometry of curvature is more intricate; see, for instance, the comparison between the capacities of four-paraboloid and spherical pairs discussed in Section 3.1.

**Figure 3:**
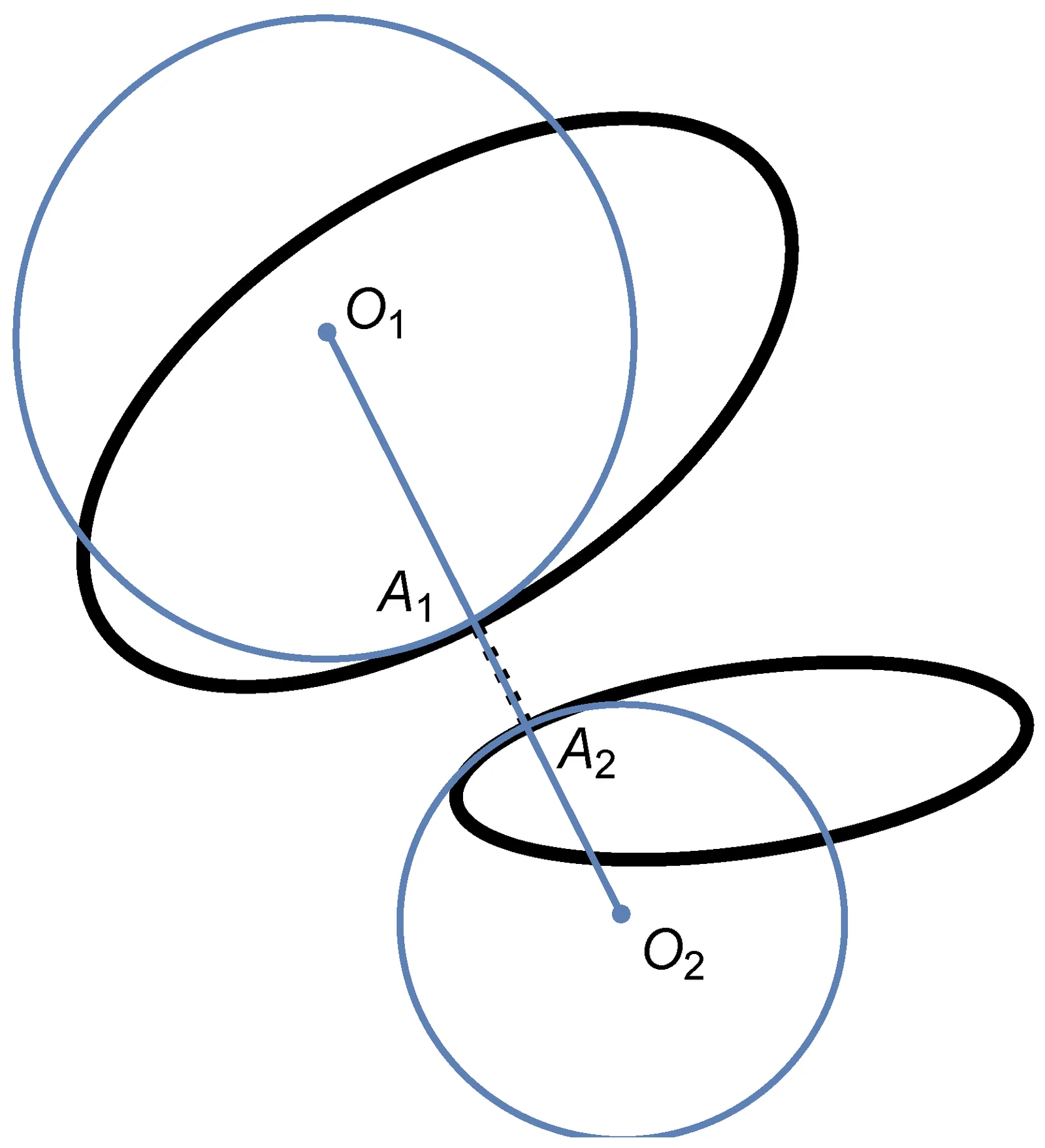
Two elliptic inclusions bounded by solid lines. The shortest distance between the ellipses is connected by the dotted line $A_{1}A_{2}$. Blue circles touch the ellipses at $A_{1}$ and $A_{2}$. Their radii $|O_{1}A_{1}|$ and $|O_{2}A_{2}|$ are the radii of curvature of the ellipses at points $A_{1}$ and $A_{2}$.

To illustrate this idea, consider the network approximation for a densely packed array of ellipses. We begin by computing the capacity of two ellipses using Kolpakov’s Formula (9). This capacity coincides with that of two disks whose radii equal the curvature radii $r_{k}$ and $r_{m}$ at the closest points on the ellipses, as depicted in Figure 3. Here, $\delta_{km}=\left|A_{1}A_{2}\right|$, $r_{k}=\left|O_{1}A_{1}\right|$ and $r_{m}=\left|O_{2}A_{2}\right|$ are the corresponding curvature radii at the points of closest distance.

A Delaunay-type graph and network approximation can be used to determine the effective conductivity of densely packed elliptical inclusions, as described in Section 3.2. The standard Voronoi tessellation does not apply here. An analog of a Delaunay graph can be constructed by minimal-distance connections between ellipses.

## 4. Elliptic Inclusions

Following the strategy [71,93], we go ahead and use expansion of the effective constants in the concentration *f* near the point f=0. The most general results on the effective conductivity of plane elliptic inclusions were obtained in [76,94] for general shapes. We use analytical formulas from [76] and derive new asymptotic formulas for densely packed ellipses. The presented results for random structures can be considered as an extension of the formulas [95,96,97] established for regular structures by Rayleigh’s method [98] and by series representations of elliptic functions [54,55,99,100].

**Figure 4:**
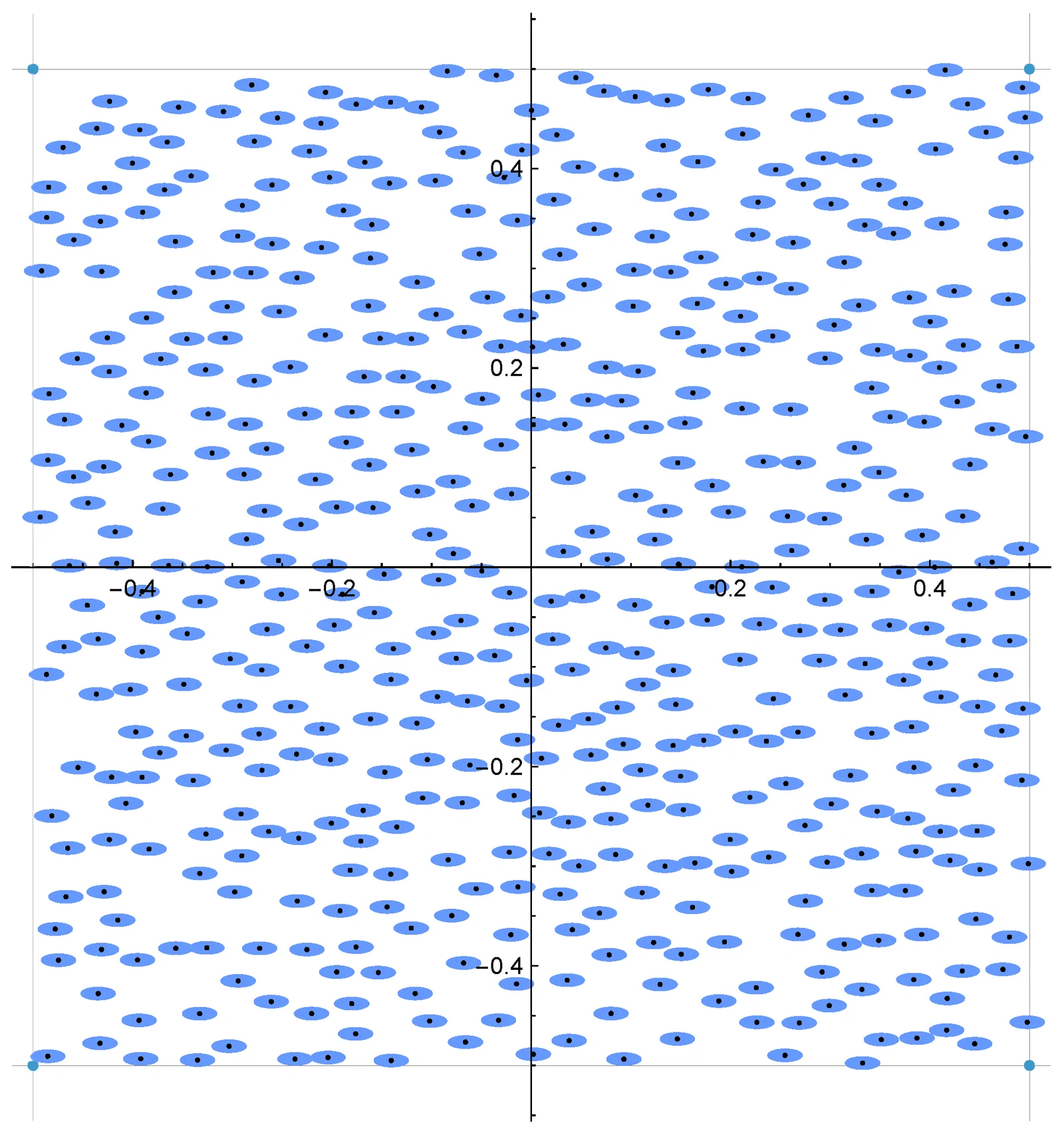
Unidirectional elliptic inclusions.

**Figure 5:**
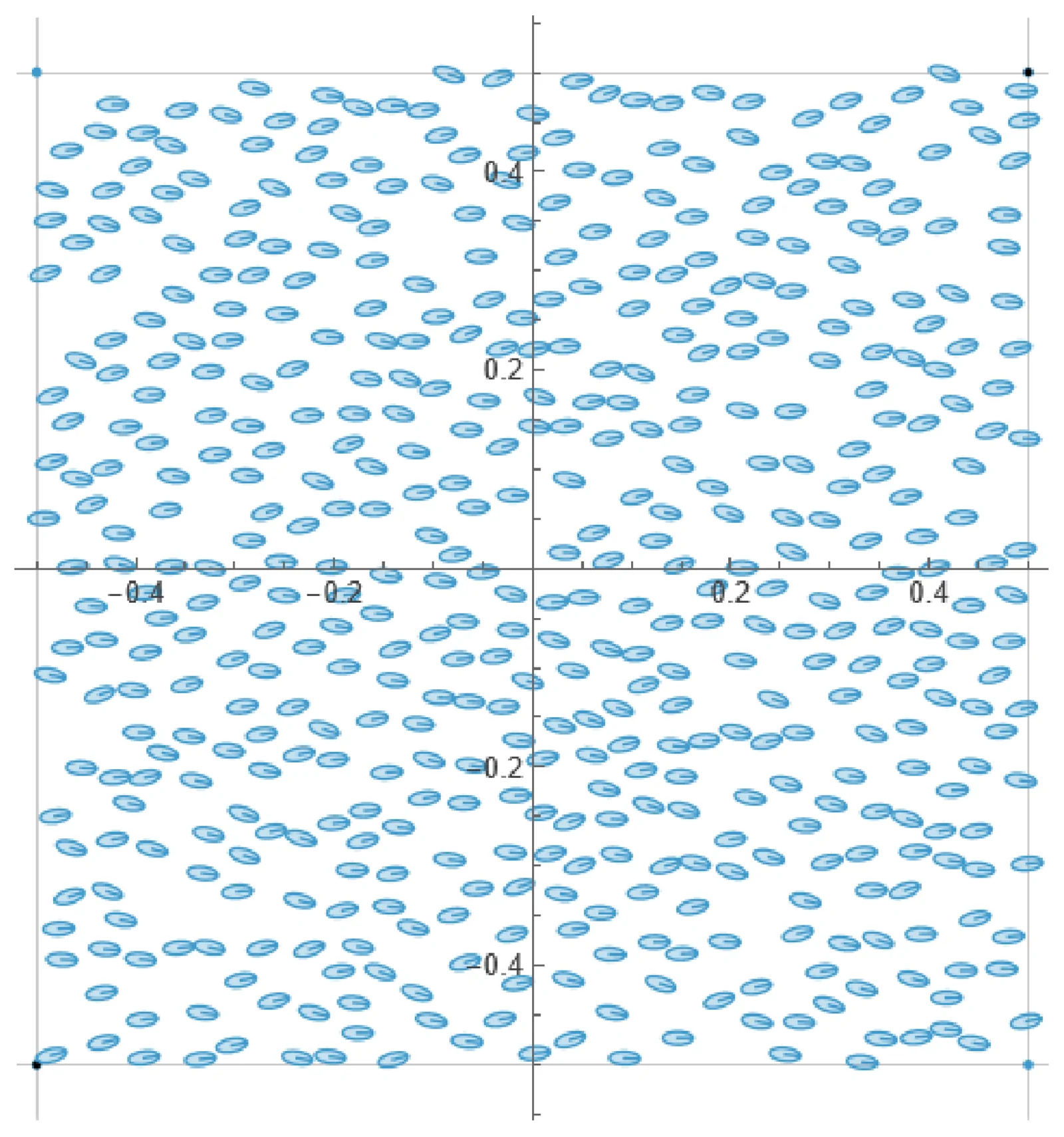
Randomly inclined elliptic inclusions.

**Figure 6:**
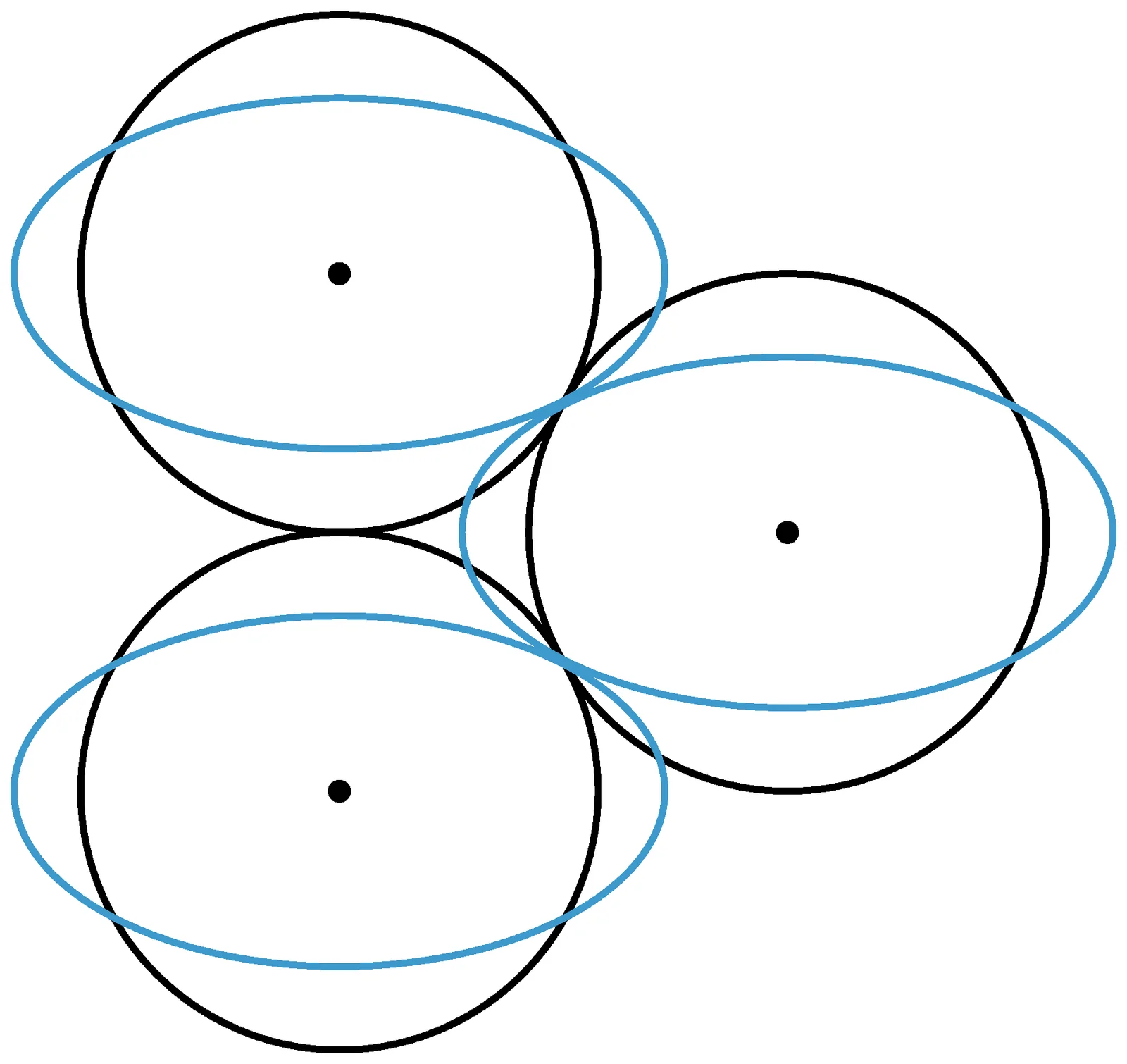
Three circles and three ellipses from the unit hexagonal array generated by their common centers.

Consider non-overlapping $D_{k}$ (k=1,2,…,N) in the torus topology bounded by equal ellipses $L_{k}$, as shown in Figure 4 and Figure 5. Let $a_{k}\in Q$ (k=1,2,…,N) denote the centers of ellipses. It is convenient to consider a parametric equation of ellipses in the complex form:

(15) $$x_{1}+ix_{2}\equiv t=a_{k}+re^{i\theta_{k}}\left(e^{i\phi }+\alpha e^{-i\phi }\right),$$

where the semi-axes of the ellipse hold r(1+α) and r(1−α) with 0≤α<1. The angle $\theta_{k}$ is the inclination of ellipse to the axis $Ox_{1}$. Similar to the case of circular inclusions, *D* denotes the host perforated domain, i.e., the complement of the closure of all the ellipses in *Q*. Figure 6 illustrates the effect of percolation when the circles transform into ellipses.

First, introduce the classic static moments used in various fields of applied mathematics to describe the geometric properties of domains [101]. It will be convenient to use the static moments in the complex form:

(16) $$s_{qk}=\int_{D_{k}}{\left(t-a_{k}\right)}^{q}\;dx_{1}dx_{2},\;k=1,2,\ldots ,N,\;q=0,1,\ldots .$$

Introduce the dimensionless static moments:

(17) $$s_{qk}^{\left(0\right)}=s_{qk}s_{0k}^{-\frac{q}{2}-1}.$$

Using Green’s formula and the parametrization (15), calculate the integral (17) for some *q*:

(18) $$s_{0k}^{\left(0\right)}=1,\;s_{2k}^{\left(0\right)}=\frac{\alpha e^{2i\theta_{k}}}{\pi \left(1-\alpha^{2}\right)},\;s_{4k}^{\left(0\right)}=\frac{2\alpha^{2}e^{4i\theta_{k}}}{\pi^{2}{\left(1-\alpha^{2}\right)}^{2}},\;s_{1k}^{\left(0\right)}=s_{3k}^{\left(0\right)}=0.$$

Introduce also the designations $E_{p,mk}=E_{p}\left(a_{m}-a_{k}\right)$, where $E_{p}\left(z\right)$ stands for the Eisenstein elliptic function of order p=2,3,… [73,102]. Let C denote the operator of complex conjugation. Following [71], we use the first-order structural sum:

(19) $$e_{p}=\frac{1}{N^{1+\frac{p}{2}}}\sum_{k,m=1}^{N}E_{p,mk}.$$

We will also use the higher-order structural sums [73,102]:

(20) $$e_{m_{1},\ldots ,m_{q}}=\frac{1}{N^{\alpha_{q}}}\sum_{k_{0},\ldots ,k_{q}=1}^{N}\prod_{j=1}^{q}C^{\;j+1}E_{m_{j}}\;\left(a_{k_{j-1}}-a_{k_{j}}\right),$$

where

(21) $$\alpha_{q}=1+\frac{1}{2}\sum_{j=1}^{q}m_{j}.$$

Here, it is assumed for brevity that $E_{p}\left(a_{m}-a_{k}\right)=:S_{p}$ for m≠k, where $S_{p}$ denotes the Rayleigh lattice sum [98].

Consider two-phase composites in which inclusions of conductivity $\lambda_{1}$ are embedded into the host of unit conductivity. Introduce the contrast parameter

(22) $$\varrho =\frac{\lambda_{1}-1}{\lambda_{1}+1},$$

which is equal to 1 for perfectly conducting inclusions.

We will use formulas [75] ((77)–(83)) for the components of the normalized effective conductivity tensor $\lambda_{ij}$:

(23) $$\lambda_{11}-i\lambda_{12}=1+2\varrho f+2\varrho^{2}\left(J+L+V\right)+{O(|\varrho |}^{3}f^{4}),$$

where

(24) $$J=\frac{1}{4\pi }\sum_{k=1}^{N}\int_{L_{k}}d\bar{t}\int_{L_{k}}\frac{\bar{\tau }}{\tau -t}\;d\tau ,$$

(25) $$L=\frac{1}{\pi }\sum_{k,m=1}^{N}|D_{k}\left|\right|D_{m}|E_{2,mk}\;,$$

and

(26) $$V=V^{\left(2\right)}+V^{\left(3\right)},$$

with

(27) $$\begin{array}{c} V^{\left(2\right)}=\frac{3}{\pi }\sum_{k,m=1}^{N}|D_{k}\left|\right|D_{m}|E_{4,mk}\left(|D_{k}|s_{2k}^{\left(0\right)}+\left|D_{m}\right|s_{2m}^{\left(0\right)}\right), \end{array}$$

(28) $$\begin{array}{c} V^{\left(3\right)}=\frac{4}{\pi }\sum_{k,m=1}^{N}|D_{k}\left|\right|D_{m}|E_{5,mk}\;\left(|D_{k}{|}^{\frac{3}{2}}s_{3k}^{\left(0\right)}-{\left|D_{m}\right|}^{\frac{3}{2}}s_{3m}^{\left(0\right)}\right). \end{array}$$

In the considered case of equal, randomly inclined ellipses, the above general formulas can be reduced. Substituting the value $|D_{k}|=\pi r^{2}\left(1-\alpha^{2}\right)$, the area of domain $D_{k}$ bounded by the ellipse $L_{k}$ into the term (25) we express this terms by the structural sum:

(29) $$L=\frac{f^{2}}{\pi }e_{2}.$$

Using Formula (18), we obtain $V^{\left(3\right)}=0$ and

(30) $$V\equiv V^{\left(2\right)}=-\frac{6\alpha^{2}f^{3}{\left(1-\alpha^{2}\right)}^{2}}{\pi^{3}N^{3}}\sum_{k=1}^{N}\sum_{m=1}^{N}\left(e^{4i\theta_{k}}+e^{4i\theta_{m}}\right)E_{4,k,m}.$$

The integral (24) for the considered elliptical inclusions becomes

(31) $$J=\frac{\alpha f}{N}\sum_{k=1}^{N}e^{2i\theta_{k}}.$$

**Example 1.**  *Let all the ellipses be unidirectional, i.e., $\theta_{k}=0$ (k=1,2,…,N), as shown in Figure 4. Then,*

(32) $$V=-\frac{12\alpha^{2}f^{3}{\left(1-\alpha^{2}\right)}^{2}}{\pi^{3}}e_{4},\;J=\alpha f,$$

*and* (23) *becomes*

(33) $$\lambda_{11}-i\lambda_{12}=1+2\varrho f+2\alpha f\varrho^{2}+\frac{2f^{2}\varrho^{2}e_{2}}{\pi }-\frac{24\alpha^{2}f^{3}\varrho^{2}{\left(1-\alpha^{2}\right)}^{2}e_{4}}{\pi^{3}}+{O(|\varrho |}^{3}f^{4}).$$

*For the structure simulated in Figure 4 and Figure 5, we obtain*

(34) $$e_{2}=3.1398-0.004965i,\;e_{4}=-0.1261+0.2739i,\;e_{22}=12.7101,\;e_{33}=-6.43104.$$

*Then,* (33) *for α=0.4 yields*

(35) $$\lambda_{11}=1+2\varrho f+0.8f\varrho^{2}+1.9988f^{2}\varrho^{2}+0.02213f^{3}\varrho^{2}+{O(|\varrho |}^{3}f^{4}).$$

If the locations of the ellipses change, the structural sums (34); hence, Formula (35) also changes. For the hexagonal (triangular) array, we have [5]

(36) $$e_{2}=\pi ,\;e_{4}=0,\;e_{22}=\pi^{2},\;e_{33}=0,$$

and

(37) $$\lambda_{11}=1+2\varrho f+0.8f\varrho^{2}+2f^{2}\varrho^{2}+{O(|\varrho |}^{3}f^{4}).$$

The approximation (35) is constructed near point f=0.35 for the configuration displayed in Figure 4. The RSA method [71] provides unstable results for $e_{4}$. The real part of $e_{4}$ takes a value from −150 to 0. At the same time, the computations of $e_{2}$, $e_{22}$, and $e_{33}$ are stable and yield results near the values (33) for various configurations and other concentrations near 0.45.

### 4.1. Critical Index—General Scheme

The effective conductivity response of an isotropic composite with infinitely conductive (superconductive) circular inclusions can be approximated through polynomial expansions in the volume fraction *f*.

For every configuration, the normalized effective conductivity $\lambda_{e}\left(f\right)$ was represented by a truncated series [5,13]:

(38) $$\lambda_{e,k}\left(f\right)=\sum_{n=0}^{k}b_{n}f^{n},$$

where the coefficients $b_{n}$ were computed exactly from structural sums [103]. These sums encode the geometric correlations of the disk arrangement and therefore depend on the protocol used to generate the microstructure. In isotropic composites, one has $b_{0}=1$ and $b_{1}=b_{2}=2$, while higher-order coefficients vary with the protocol.

For instance, in the two-dimensional case of superconducting disks randomly distributed in the conducting host, as the concentration approaches the maximally possible value $f_{c}=\frac{\pi }{\sqrt{12}}\approx 0.9069$, the effective conductivity according to [5,24,73] has the following behavior:

(39) $$\lambda_{e}\left(f\right)∼B{\left(f_{c}-f\right)}^{-s},$$

where s≈1.3 is the critical exponent and *B* is the corresponding amplitude. Both quantities are dimensionless because the host conductivity is normalized to unity.

Within the framework of continuous percolation, it is crucial to rigorously distinguish the true percolation threshold from the purely geometric limits of the composite structure:

- **Jamming Threshold ($f_{J}$):** The maximal packing fraction dictated by the specific generation protocol, corresponding to a kinetically arrested state where the dispersed inclusions form a rigid, mechanically stable structure and no further compression is possible without particle overlap.
- **Contact Percolation Threshold ($f_{\mathrm{cp}}$):** The geometric volume fraction at which an unbroken topological chain of strictly zero-distance (touching) inclusions first spans the system. While $f_{cp}$ often coincides with $f_{J}$ or $f_{c}$ in ideal hard-disk protocols, it remains a purely structural metric.
- **True Transport Threshold ($f_{c}$):** The critical singularity where the macroscopic effective conductivity diverges. Some exceptions are given in Section 3.1. In densely packed random composites, transport is dominated by the highly intensified fluxes across the narrow, non-touching gaps between adjacent inclusions. Consequently, the true transport threshold $f_{c}$, which is extracted here via the asymptotic resummation of our structural sums, is governed by these gap conductances rather than binary geometric contacts, making it a physically distinct parameter from $f_{cp}$ and $f_{J}$.

By utilizing high-order resummation schemes (such as Padé approximants $P_{m,n}$), our analysis explicitly targets the true transport threshold $f_{c}$, capturing how microstructural gap configurations control the critical divergence independently of isotropic random close-packing limits.

In the cases of various composites considered in the current review the critical index is considered as unknown. To determine *s*, the truncated series are extrapolated toward the critical regime using self-similar renormalization techniques. These include Borel-type transformations [104], iterated-root approximants [5], and optimization criteria such as minimal-difference and minimal-sensitivity conditions [105,106,107,108]. Together, these tools allow the dilute-regime coefficients to be linked to the asymptotic divergence near $f_{c}$ without introducing additional fitting parameters. When the threshold is unknown as well, we resort to the Padé approximants [109,110], and estimate $f_{c}$ as corresponding to simple poles.

We apply to the original truncated power series written down as (38), the Borel-type transformation of the following form:

(40) $$\phi_{k,b}\left(f\right)=\sum_{n=0}^{k}\frac{b_{n}}{{\left(\Gamma (1+n)\right)}^{b}}\;f^{n}\;$$

to improve its convergence [24,111].

Parameter *b* stands for the discrete number of Borel iterations. The case of b=0 returns us to the original polynomial approximations, while setting b=1 corresponds to the classical Borel transformation [104].

The transformation $f=\frac{f_{c}z}{1+z}$ is applied to the Borel-transformed series (40). The iterated root approximants

(41) $$\phi_{k,b}^{*}={\left({\left({\left({\left(1+A_{1,b}z\right)}^{2}+A_{2,b}z^{2}\right)}^{3/2}+A_{3,b}z^{3}\right)}^{4/3}+\ldots +A_{k,b}z^{k}\right)}^{s_{k,b}/k}\;$$

are applied to such transformed series. Here, $z=\frac{f}{f_{c}-f}$, while parameters $A_{k,b}$ are determined by asymptotically matching formulas for the polynomial expressions and approximants. The critical index $s\approx s_{k,b}$. The index *s* will be treated as a parameter to be determined from some plausible additional conditions.

The amplitudes at $f\to f_{c}$ are approximated as follows [24]:

(42) $$B_{k,b}\left(s\right)=f_{c}^{s}C_{k,b}\left(s\right){\left(\Gamma \left(1+s\right)\right)}^{b}\;,$$

where

(43) $$C_{k,b}\left(s\right)={\left({\left({\left(A_{1,b}{\left(s\right)}^{2}+A_{2,b}\left(s\right)\right)}^{3/2}+A_{3,b}\left(s\right)\right)}^{4/3}+\ldots +A_{k,b}\left(s\right)\right)}^{s_{k,b}/k}\;.$$

Marginal amplitudes $C_{k,b}\left(s\right)$ are expressed in the limit z→∞ from the expression for iterated roots [5,73]. The derivation of self-similar root approximants is inspired by general ideas of renormalization group [112,113,114,115].

### 4.2. Critical Index for Slightly Inclined Ellipses

Let all the ellipses be randomly inclined, as shown in Figure 5. It is assumed that the random variables $\theta_{k}$ are uniformly distributed in the interval (−Θ,+Θ). Then,

(44) $$V=-\frac{6\alpha^{2}f^{3}{\left(1-\alpha^{2}\right)}^{2}}{\pi^{3}}e_{4},\;J=\alpha f,$$

and (23) becomes

(45) $$\lambda_{11}-i\lambda_{12}=1+2\varrho f+\frac{\alpha f\varrho^{2}\sin2\Theta }{\Theta }+\frac{2f^{2}\varrho^{2}}{\pi }e_{2}-\frac{6\alpha^{2}f^{3}\varrho^{2}{\left(1-\alpha^{2}\right)}^{2}\sin4\Theta }{\pi^{3}\Theta }e_{4}+{O(|\varrho |}^{3}f^{4}).$$

For the structure simulated in Figure 5, we take Θ=0.1π. Then, (45) for α=0.4 yields

(46) $$\lambda_{11}=1+2f\varrho +0.7484f\varrho^{2}+1.9989f^{2}\varrho^{2}+0.016749f^{3}\varrho^{2}+{O(|\varrho |}^{3}f^{4}).$$

For the hexagonal (triangular) array, we have

(47) $$\lambda_{11}=1+2f\varrho +0.7484f\varrho^{2}+2f^{2}\varrho^{2}+{O(|\varrho |}^{3}f^{4}).$$

The critical concentration holds $f_{c}=0.7618$ for (47). It can be accepted for (46).

In the case of ϱ=1, in order to find the approximations for the index, a minimal sensitivity condition [24] is employed:

(48) $$\frac{\partial B_{2,0}\left(s\right)}{\partial s}=0,\;s=s_{2,0},$$

It does not include the Borel transformation. The critical index *s* is again the principal quantity of interest, and the effective conductivity is found by solving Equation (48). The solution to (48) can be found:

$$k=2,\;\;\;\;s=s_{2,0}=0.50274.$$

To find the approximations for the index, a minimal difference condition could be imposed [24]:

(49) $$B_{2,0}\left(s\right)-B_{1,0}\left(s\right)=0,$$

and $s=s_{2,0}=s_{1,0}$ for k=2. In such a case, condition (49) does not include the Borel transformation at all. Thus, Equation (49) depends on the critical index by itself and allows us to find it simultaneously. The solution to (49) can be found as follows: $k=2,\;\;\;\;s=s_{2,0}=0.70141.$

By taking their average, we estimate s≈0.60208±0.09934. The notation $s=\bar{s}\pm \Delta s$ does not denote a statistical confidence interval. Here, $\bar{s}$ is the arithmetic mean of the critical exponents obtained from different resummation procedures, and Δs is one half of the spread between these estimates. Therefore, Δs quantifies methodological dispersion associated with the reconstruction procedure rather than statistical uncertainty arising from ensemble averaging.

### 4.3. Critical Index for Randomly Inclined Ellipses

Let $\Theta =\frac{\pi }{2}$. This case corresponds to a macroscopically isotropic case. Combining the results of [76,94], we arrive at the following formula:

(50) $$\begin{array}{c} \lambda_{e}=1+2f\varrho \frac{1}{1-\alpha^{2}\varrho^{2}}+2f^{2}\varrho^{2}\frac{1+\alpha^{2}}{1-\alpha^{2}}\frac{1}{{\left(1-\alpha^{2}\varrho^{2}\right)}^{2}}+2f^{3}\varrho^{3}\frac{1}{\pi^{2}}{\left(\frac{1+\alpha^{2}}{1-\alpha^{2}}\right)}^{2}\frac{1}{{\left(1-\alpha^{2}\varrho^{2}\right)}^{3}}e_{22}\\ -4f^{4}\varrho^{3}\frac{{\left(\alpha^{2}+1\right)}^{3}}{\pi^{3}{\left(1-\alpha^{2}\right)}^{3}{\left(1-\alpha^{2}\varrho^{2}\right)}^{3}}e_{33}+\frac{2{\left(\alpha^{2}+1\right)}^{3}f^{4}\varrho^{4}}{\pi^{2}{\left(1-\alpha^{2}\right)}^{3}{\left(1-\alpha^{2}\varrho^{2}\right)}^{4}}\left(2e_{22}-\pi^{2}\right)+O\left(f^{5}\right). \end{array}$$

**Figure 7:**
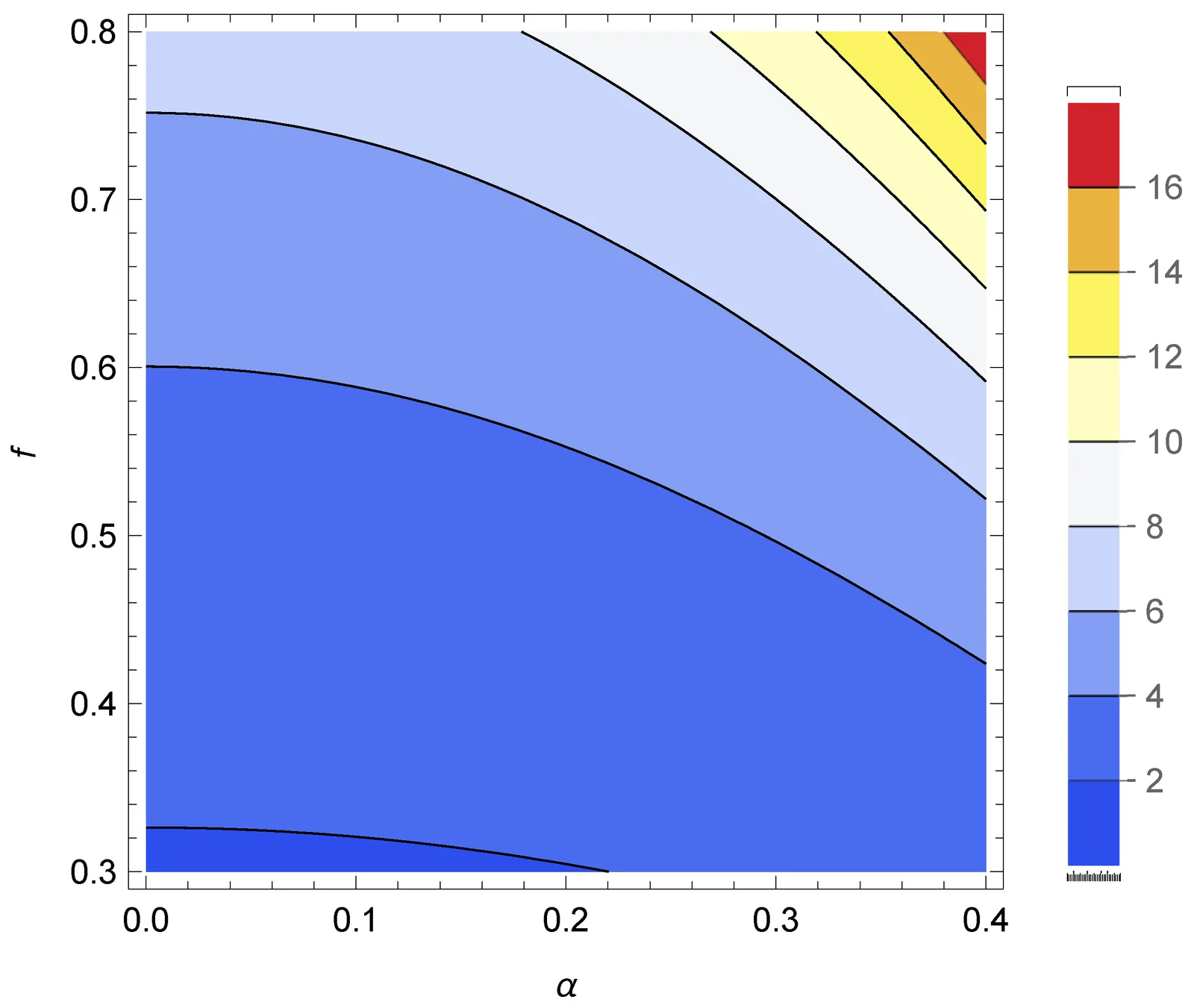
The dependence of the effective conductivity calculated with (50) on α and *f* for ϱ=1.

An illustration of Formula (50) is presented in Figure 7.

In the case (34), the Formula (50) for α=0.4 and ϱ=1 becomes

(51) $$\begin{array}{c} \lambda_{e}=1+2.38095f+3.91426f^{2}+8.28703f^{3}+20.3548f^{4}+O\left(f^{5}\right). \end{array}$$

The critical concentration is the same as in the previous example, $f_{c}=0.7618$. The structural sum $e_{4}=0$ for macroscopically isotropic composites. Expression (51) contains stable coefficients.

In the case of polynomial (51) with ϱ=1, in order to find the approximations for the index *s* for the effective conductivity $\lambda_{e}$, a familiar minimal sensitivity condition can be imposed [24,73]:

(52) $$\frac{\partial B_{k,b}\left(s\right)}{\partial s}=0,\;s=s_{k,b},$$

It does include the Borel transformation. The critical index *s* is again the principal quantity of interest, and the effective conductivity is found by solving Equation (52).

For polynomial (51), the numerically convergent solution to the minimal sensitivity condition (52) can also be found for various *k* and b=0:

$$k=2,\;\;\;\;s=s_{2,0}=0.56448;$$

$$k=3,\;\;\;\;s=s_{3,0}=0.62729;$$

$$k=4,\;\;\;\;s=s_{4,0}=0.70553.$$

Good numerical convergence is again evident here, and the last value of s≈0.7055.

For polynomial (51), the numerically convergent solution to the minimal sensitivity condition (52) can also be found for various *k* and b=1:

$$k=2,\;\;\;\;s=s_{2,1}=0.58888;$$

$$k=3,\;\;\;\;s=s_{3,1}=0.61901;$$

$$k=4,\;\;\;\;s=s_{4,1}=0.64692.$$

Good numerical convergence is again evident here, and the last value of s≈0.647.

For polynomial (51), the numerically convergent solution to the minimal sensitivity condition (52) can also be found for various *k* and b=2:

$$k=2,\;\;\;\;s=s_{2,2}=0.70751;$$

$$k=3,\;\;\;\;s=s_{3,2}=0.68367;$$

$$k=4,\;\;\;\;s=s_{4,2}=0.68954.$$

The best numerical convergence is to the last value of s≈0.6895. Averaging the best estimates for k=4 and b=0,1,2 yields

(53) s=0.68066±0.02474.

The considered case of randomly inclined ellipses requires a comparison with the known formulas for a macroscopically isotropic composite. The previously derived formulas hold in the first-order approximation in *f* [94]. Ref. [116] at the end of §22 contains a numerically derived table of the normalized effective viscosity η≈1+Af of randomly distributed spheroids for several values of the shape factor *A*. In particular, spherical particles give the Eisenstein formula with $A=\frac{5}{2}$. In the considered 2D case, the shape factor takes the form $A=\frac{2\varrho }{1-\alpha^{2}\varrho^{2}}$ as follows from (50). Hence, Formula (50) represents a higher-order approximation that enables the determination of the critical concentration and the associated critical exponent as functions of the parameter α. It should be emphasized that the empirical results reported in [117] (see, for instance, Formula (16)) are inconsistent with the classical results of [94,116], as well as with Equation (50).

## 5. Extension to Phase Polydisperse Composites

The asymptotic formulas for the effective constants discussed above can be extended to phase composites. Following [5,73], consider *N* mutually disjoint disks per the unit square cell *Q* with different conductive or elastic constants.

Consider perfectly conducting non-overlapping disks $D_{k}=\{|z-a_{k}\left|<r_{k}\right\}$ (k=1,2,…,N) embedded in a host of normalized unit conductivity. Introduce the polydispersity parameters

(54) $$\varrho_{k}^{\prime }={\left(\frac{r_{k}}{r_{\mathrm{ref}}}\right)}^{2},$$

where $r_{\mathrm{ref}}^{2}=\frac{1}{N}\sum_{k=1}^{N}r_{k}^{2}$. In [5,73], the reference radius $r_{\mathrm{ref}}$ was taken as the $\max_{j}r_{j}$. We introduce the following fractions:

(55) $$f_{k}=\pi r_{k}^{2}=\pi r_{\mathrm{ref}}^{2}\varrho_{k}^{\prime },\;f=\sum_{k=1}^{N}f_{k}=\pi r_{\mathrm{ref}}^{2}N.$$

**Figure 8:**
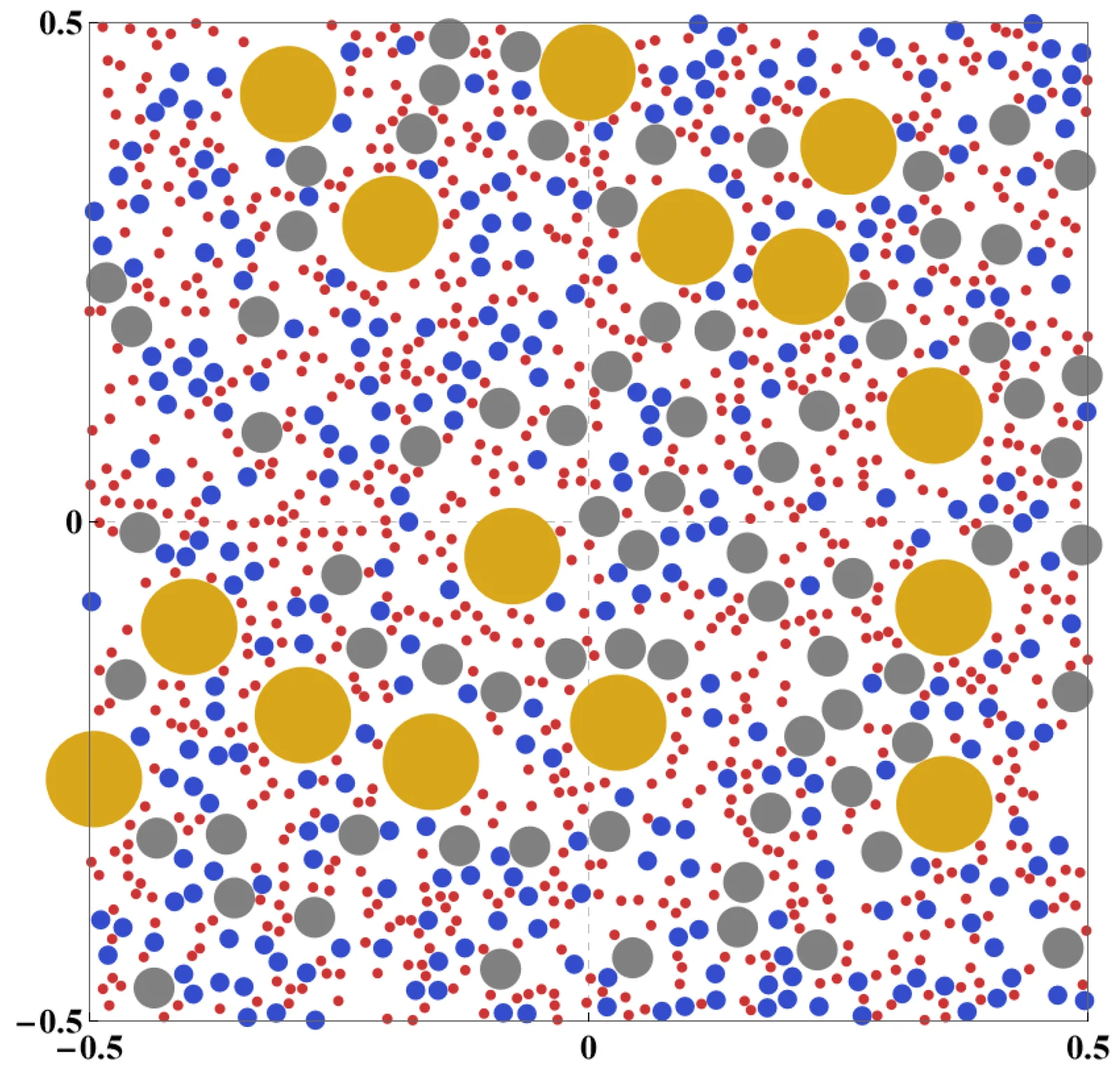
Structure A. In total, there are 1290 random non-overlapping circular inclusions of four different radii $\left(r_{1}=0.048,\;r_{2}=0.021,\;r_{3}=0.010,\;r_{4}=0.005\right)$ in the representative unit square cell. Different colors mean different radii.

**Figure 9:**
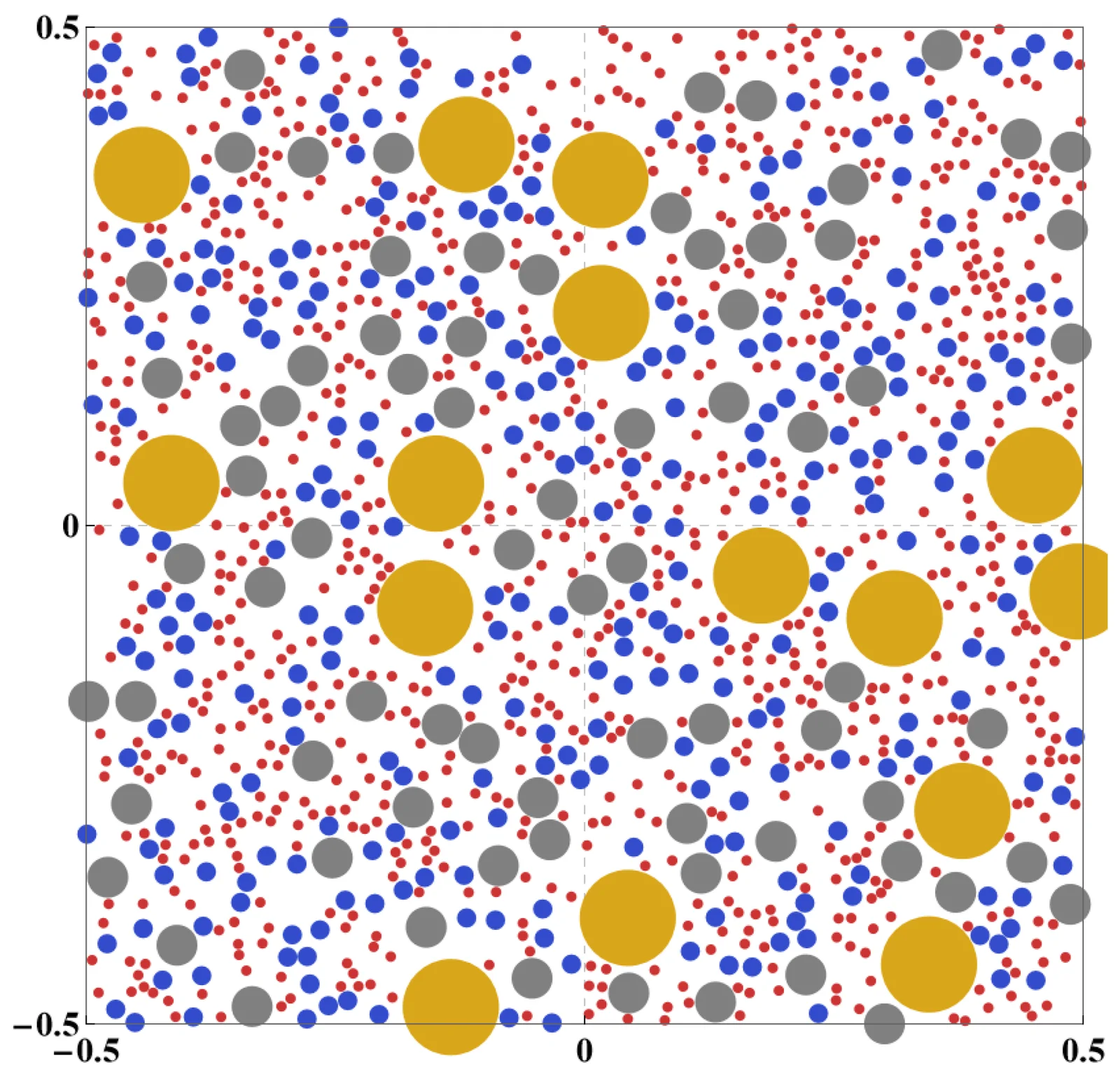
Structure B. In total, there are 1290 random non-overlapping circular inclusions of four different radii $\left(r_{1}=0.048,\;r_{2}=0.021,\;r_{3}=0.010,\;r_{4}=0.005\right)$ in the representative unit square cell.

Figure 8 and Figure 9 show two variants, denoted as A and B, which are simulated by the same rules of a two-dimensional random composite microstructure consisting of circular non-overlapping inclusions with four distinct fixed radii. The centers of inclusions are randomly distributed within the unit square, doubly periodically tiling the whole plane. The total number of particles is N=1290, divided into four groups corresponding to different size scales. The radii are chosen to ensure prescribed area fractions for each group, resulting in a multi-scale heterogeneous structure. Periodicity conditions (4) are taken into account when enforcing the non-overlap constraint.

We introduce the polydispersity structural sums generalizing the monodispersed case (20):

(56) $$h_{m_{1},\ldots ,m_{q}}=\frac{1}{N^{\alpha_{q}}}\sum_{k_{0},\ldots ,k_{q}=1}^{N}\left[\prod_{j=0}^{q}{\left(\varrho_{k_{j}}^{\prime }\right)}^{t_{j}}\right]\prod_{j=1}^{q}C^{\;j+1}E_{m_{j}}\;\left(a_{k_{j-1}}-a_{k_{j}}\right),$$

where $\alpha_{q}$ is provided by (21),

(57) $$t_{0}=1,\;t_{j}=m_{j}-t_{j-1},\;j=1,\ldots ,q,\;t_{q}=1.$$

For instance,

(58) $$h_{2}=\frac{1}{N^{2}}\underset{k_{0}=1}{\sum^{N}}\underset{k_{1}=1}{\sum^{N}}\varrho_{k_{0}}^{\prime }\varrho_{k_{1}}^{\prime }E_{2}\left(a_{k_{0}}-a_{k_{1}}\right),$$

(59) $$h_{2,2}=\frac{1}{N^{3}}\underset{k_{0}=1}{\sum^{N}}\underset{k_{1}=1}{\sum^{N}}\underset{k_{2}=1}{\sum^{N}}\varrho_{k_{0}}^{\prime }\varrho_{k_{1}}^{\prime }\varrho_{k_{2}}^{\prime }E_{2}\left(a_{k_{0}}-a_{k_{1}}\right)\;\bar{E_{2}\left(a_{k_{1}}-a_{k_{2}}\right)},$$

and

(60) $$h_{3,3}=\frac{1}{N^{4}}\sum_{k_{0},k_{1},k_{2}=1}^{N}\varrho_{k_{0}}^{\prime }{\left(\varrho_{k_{1}}^{\prime }\right)}^{2}\varrho_{k_{2}}^{\prime }E_{3}\;\left(a_{k_{0}}-a_{k_{1}}\right)\bar{E_{3}\;\left(a_{k_{1}}-a_{k_{2}}\right)}.$$

The normalized effective conductivity of macroscopically isotropic polydisperse composites can be calculated following [5,73]:

(61) $$\lambda_{e}=1+2f+2f\left(B_{1}f+B_{2}f^{2}+B_{3}f^{3}\right)+\ldots$$

where the first few coefficients are written below:

(62) B1=1πh2,B2=1π2h22,B3=1π3−2h33+h222,B4=1π43h44−4h332+h2222,B5=1π5−4h55+6(h442+2h343)−2(h2332+2Reh2233)+h22222,B6=1π6[5h66−8(h255+3h354)+6Reh2244+12Reh2343+4h3333+3h2442−4(h22233+h22332)+h222222],B7=1π7[−6h77+10(h266+4h365+3h464)−2(2h2255+6h2354+6h2453+2h2552+3h3344+9h3443+6h3542+3h4433+6h4532+2h5522)+2(3Reh22244+6Reh22343+3Reh22442+4Reh23333+3h23432+2h33233)−2(2h222233+2h222332+h223322)+h2222222].B8=1π8[7h88+Re(6h244222+8h333322+6h442222)+16Reh5533−2(h2223322+2h2233222+4h2332222+4h3322222)+(6h222343+6h223432+3h224422+4h233233+4h233332+6h234322+4h332233+4h332332+6h343222)−(12h22354+12h22453+6h23344+18h23443+12h23542+6h24433+12h24532+8h25522+12h33343+6h33442+12h34333+18h34432+12h35422+12h44233+6h44332+12h45322+8h55222)+(20h2365+30h2464+20h2563+5h2662+36h3454+48h3553+20h3652+9h4444+36h4543+30h4642+20h5632+10h6622)−(30h376+60h475+60h574+30h673+12h772)+h22222222].

### Discussion

Figure 8 and Figure 9 display two distinct stochastic realizations (variants A and B) of a two-dimensional, randomly dispersed composite generated under identical macroscopic constraints. While these structures share the exact same global parameters, their localized geometric differences produce measurable variations in their critical asymptotic behavior. Specifically, these figures demonstrate the following physical and methodological features:

- **Identical Macroscopic Constraints:** Both structures are built using the exact same generation protocol, containing N=1290 non-overlapping circular inclusions. They are partitioned into four identical size scales ($r_{1}=0.048$, $r_{2}=0.021$, $r_{3}=0.010$, and $r_{4}=0.005$) and evaluated near an initial concentration of f=0.38.
- **Microscopic Structural Variability:** Because the inclusion centers are distributed randomly across the unit square cell, the local spatial arrangements and the specific narrow gaps formed between inclusions differ entirely between Structure A and Structure B.
- **Divergence in Polynomial Coefficients:** Methodologically, this purely microscopic geometric variance causes the structural sums for each variant to differ. This results in distinct coefficients for their respective effective conductivity polynomials (e.g., Structure A yields a second-order term of $2.04709f^{2}$, whereas Structure B yields $1.99552f^{2}$).
- **Variation in Critical Thresholds ($f_{c}$):** Applying non-diagonal Padé approximants to these distinct polynomials yields different estimates for the critical percolation threshold. Structure A yields an upper bound of $f_{c}\approx 0.56757$ (via $P_{3,4}$), while Structure B yields a slightly higher bound of $f_{c}\approx 0.58963$ (via $P_{3,4}$).
- **Variation in Critical Exponents (s):** The physical consequence of this sample-to-sample threshold variation is a shift in the estimated critical exponent governing the singularity. Structure A produces an estimated index of s≈0.38832±0.11127, whereas Structure B results in s≈0.40684±0.11486.

By presenting both figures, the manuscript visually and mathematically proves that macroscopic descriptors alone, such as total volume fraction and predetermined particle radii, are insufficient to perfectly predict the onset of percolation. The exact formation of transport pathways, and thus the critical divergence parameters $f_{c}$ and *s*, remains highly sensitive to the statistical fluctuations inherent in the localized microstructure of densely packed composites. (Figure 8 and Figure 9 display the motif of terrazzo pavement near the Kaiser Wilhelm Memorial Church in Berlin; see https://www.akg-images.fr/asset/12278747/Decorative-terrazzo-pavement-and-drain-cover-with-Kaiser-Wilhelm-Memorial-Church-motif,-Breitscheidplatz,-Berlin,-Germany (accessed on 13 October 2026)).

## 6. Effective Conductivity of Densely Packed Polydisperse Composites

The critical parameters of the simulated samples A and B shown in Figure 8 and Figure 9, respectively, are discussed in the present section. It is found that the second-order coefficient of the effective conductivity of A is very close to the isotropic value of $b_{2}=2$. Hence, we speak about unbiased estimates for the index *s*.

If it is assumed that the second-order coefficient $b_{2}=2$, then we speak about biased estimates for the index *s*.

### 6.1. Unbiased Estimates for Almost Perfect Isotropy—Structure A

The complex-valued conductivity expansion of structure A shown in Figure 8 is reduced to its real part up to $O(f^{8})$:

(63) $$\begin{array}{c} \lambda_{e}=1+2f+2.04709f^{2}+2.88935f^{3}+4.15338f^{4}+6.78494f^{5}+10.9595f^{6}+19.4464f^{7}+O\left(f^{8}\right). \end{array}$$

The imaginary part of $\lambda_{e}$ in computations is practically zero and therefore indistinguishable in the graphical representation. The statistical anisotropy of the generated RVE was estimated and discussed in [93].

The structure shown in Figure 8 is computed near the point f=0.38. This is the minimal concentration when the smallest disks of radius $r_{4}=0.005$ become touching. However, these disks have sufficient room to change their location, which can increase the critical concentration $f_{c}$. It is worth noting that the maximal geometrical concentration is not known and may be smaller than the concentration $f_{c}=\frac{\pi }{\sqrt{1}2}\approx 0.9069$ of the regular hexagonal array.

The imaginary contributions obtained in the numerical computation are discarded by taking the real part of (61).

Non-diagonal Padé approximants [109], applied in the highest approximation order to the polynomial (63), produce several sensible estimates for the critical point $f_{c}$ with the approximations $P_{3,4}$, $P_{4,3}$. The lower estimate, 0.5484, is produced by $P_{4,3}$, while the higher estimate, 0.56757, is found from $P_{3,4}$.

The approximant $P_{3,4}$ is provided in the following compact form:

(64) $$P_{3,4}\left(f\right)=\frac{3.66583(f-0.645918)(f+0.487563)(f+0.831271)}{\left(f-1.12065)(0.567567-f)(f+0.491583)(f+3.06929\right)}.$$

The physically relevant estimate of the critical concentration $f_{c}$ is identified as the nearest positive real pole of the Padé approximant within the interval 0<f<1. Since *f* represents a volume fraction, poles corresponding to f<0, f>1, or complex values have no physical interpretation in the present problem and are therefore classified as spurious. The difference between the predictions of the highest-order approximants $P_{3,4}$ and $P_{4,3}$ provides a practical estimate of the uncertainty associated with the Padé extrapolation procedure. It should be noted that all other Padé approximants of the same order produce close estimates for $f_{c}$. For instance, the approximant $P_{0,7}$ gives $f_{c}=0.580411$, $P_{1,6}$ gives $f_{c}=0.637327$, $P_{2,5}$ gives $f_{c}=0.460507$, $P_{5,2}$ gives $f_{c}=0.612863$, and $P_{6,1}$ gives $f_{c}=0.563575$. Taking the average over all seven available estimates yields the value of 0.567236. The latter is remarkably close to the estimate obtained from theoretically recommended [118] approximant $P_{3,4}$.

In such case, with the latter estimate for the threshold $f_{c}\approx 0.56757$, in order to find the approximations for the index, a minimal sensitivity condition can be imposed [24,73]:

(65) $$\frac{\partial B_{7,0}\left(s\right)}{\partial s}=0,\;s=s_{7,0},$$

It does not include the Borel transformation. The critical index *s* is again the principal quantity of interest, and the effective conductivity is found by solving Equation (65). The solution to (65) can be found as follows:

$$k=7,\;\;\;\;s=s_{7,0}=0.27704.$$

To find the approximations for the index, a minimal difference condition could be imposed [24]:

(66) $$B_{7,0}\left(s\right)-B_{6,0}\left(s\right)=0,$$

and $s=s_{7,0}=s_{6,0}$ for k=7. In such a case, condition (66) does not include the Borel transformation at all. Thus, Equation (66) depends on the critical index by itself and allows us to find it simultaneously. The solution to (66) can be found as follows:

$$k=7,\;\;\;\;s=s_{7,0}=0.49959.$$

**Figure 10:**
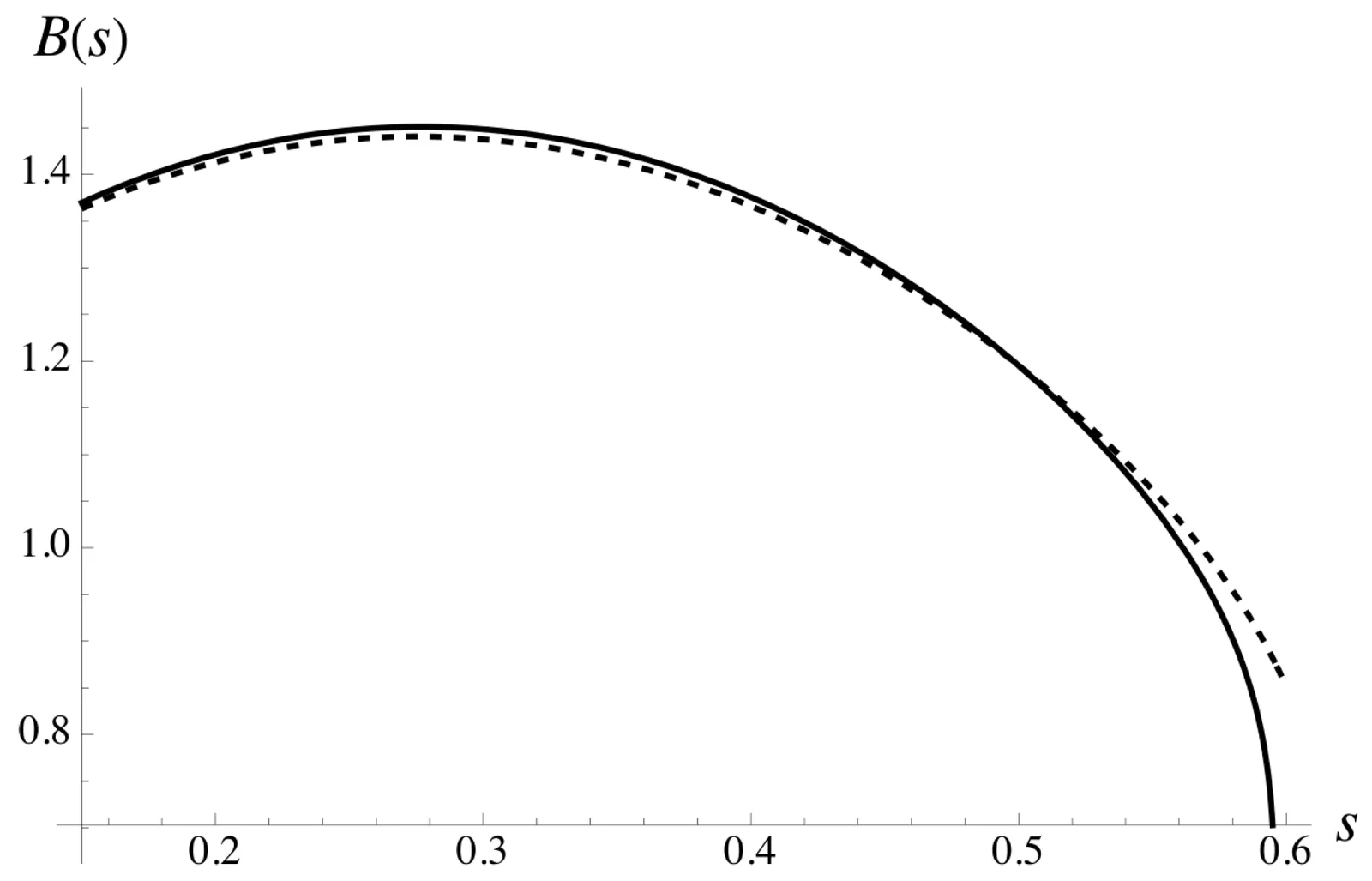
The critical amplitude $B_{7,0}\left(s\right)$ (solid), and the amplitude $B_{6,0}\left(s\right)$ (dashed), are compared dependent on the parameter *s*.

In Figure 10, the approximate amplitudes are presented for two different approximations.

By taking the average of the two different approximations, we estimate

s≈0.38832±0.11127.

The effective conductivity with such an estimated critical index is approximated by the seventh-order root approximant:

(67) $$\lambda_{e}={\left(A_{7}z^{7}+{\left(A_{6}z^{6}+{\left(A_{5}z^{5}+{\left(A_{4}z^{4}+{\left(A_{3}z^{3}+{\left(A_{2}z^{2}+{\left(A_{1}z+1\right)}^{2}\right)}^{3/2}\right)}^{4/3}\right)}^{5/4}\right)}^{6/5}\right)}^{7/6}\right)}^{0.0554736},$$

where $z=\frac{f}{f_{c}-f}$, and

$$A_{7,0}=2434.83,\;\;\;A_{6,0}=575.877,\;\;\;A_{5,0}=144.401,\;\;\;A_{4,0}=35.9878,$$

$$A_{3,0}=9.97327,\;\;\;A_{2,0}=2.77694,\;\;\;A_{1,0}=2.92323.$$

**Figure 11:**
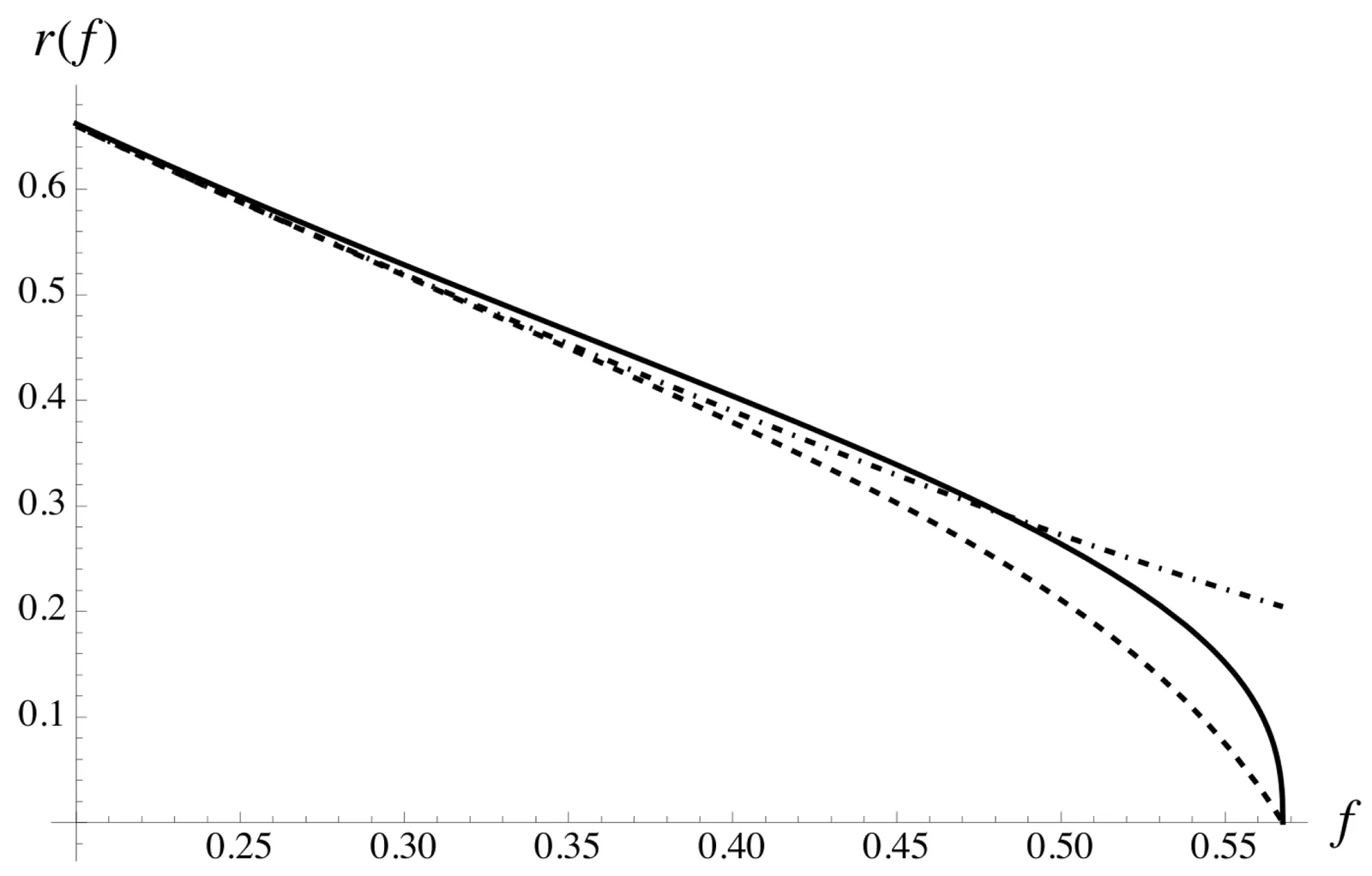
Resistance $r\left(f\right)=\lambda_{e}{\left(f\right)}^{-1}$. The final expression for the Formula (67) (solid), Padé approximant (64) (dashed); the polynomial (63) (dash-dotted) is compared dependent on the concentration *f*.

In Figure 11, the resistance $r\left(f\right)=\lambda_{e}{\left(f\right)}^{-1}$ inverse to the effective conductivity is presented for three different approximations.

### 6.2. Unbiased Estimates for Almost Perfect Isotropy—Structure B

Consider structure B of 1290 disks displayed in Figure 9 with the concentration f=0.38. The approximating polynomial was obtained as follows:

(68) $$\lambda_{e}≃1+2f+1.99552f^{2}+2.75806f^{3}+3.89348f^{4}+6.24786f^{5}+9.73745f^{6}+16.9857f^{7}.$$

Non-diagonal Padé approximants [109,110], applied at the highest approximation order to the polynomial (68), produce several sensible estimates for the critical point $f_{c}$ with the approximations $P_{3,4}$, $P_{4,3}$. The lower estimate, 0.57074, is produced by $P_{4,3}$, while the higher estimate, 0.58963, is found from $P_{3,4}$.

In such a case, with the latter estimate for the threshold $f_{c}\approx 0.58963$, in order to find the approximations for the index, a familiar minimal sensitivity condition can be imposed:

(69) $$\frac{\partial B_{7,0}\left(s\right)}{\partial s}=0,\;s=s_{7,0}.$$

It does not include the Borel transformation. The critical index *s* is again the principal quantity of interest, and the effective conductivity is found by solving Equation (69). The solution to (69) can be found as follows:

$$k=7,\;\;\;\;s=s_{7,0}=0.29198.$$

To find the approximations for the index, a minimal difference condition could be imposed:

(70) $$B_{7,0}\left(s\right)-B_{6,0}\left(s\right)=0,$$

and $s=s_{7,0}=s_{6,0}$ for k=7. In such a case, condition (70) does not include the Borel transformation at all. Thus, Equation (70) depends on the critical index by itself and allows us to find it simultaneously. The solution to (70) can be found:

$$k=7,\;\;\;\;s=s_{7,0}=0.5217.$$

By taking their average, we estimate

s≈0.40684±0.11486.

### 6.3. Unbiased Estimates for Imposed Perfect Isotropy—Random Radii

**Figure 12:**
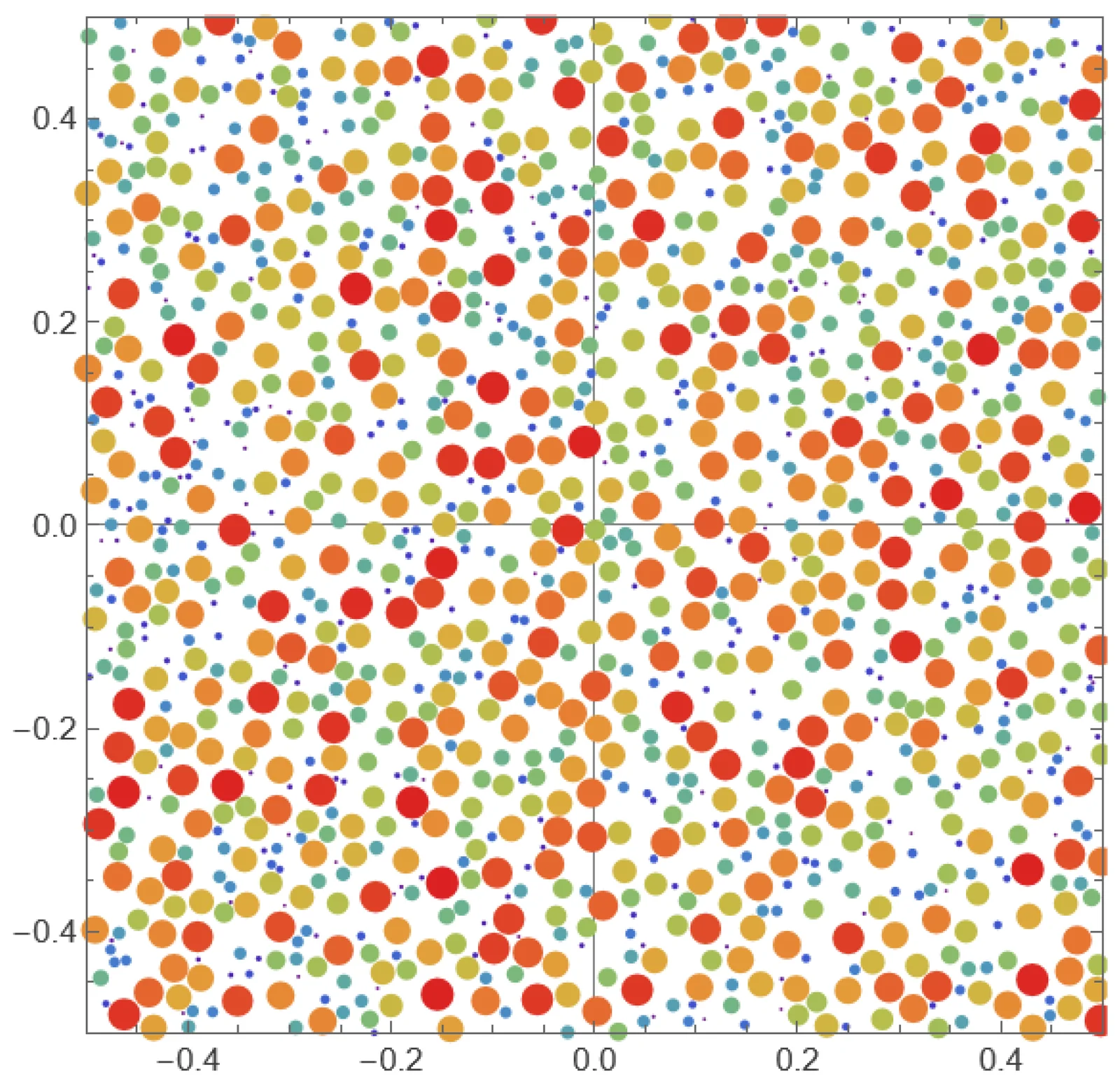
In total, there are 1290 random non-overlapping circular inclusions of random radii uniformly distributed in (0.005,0.047) with the concentration f=0.403 in the representative unit square cell. The colors correspond to the sizes of disks.

Consider the case of the disks of random radii uniformly distributed in the interval (0.005,0.047) as shown in Figure 12. The approximating polynomial is obtained as follows:

(71) $$\lambda_{e}=1+2f+1.97995f^{2}+2.47329f^{3}+3.49969f^{4}+5.61084f^{5}+9.03672f^{6}+15.4211f^{7}.$$

Non-diagonal Padé approximants applied in the highest approximation order to the polynomial (71) produce several sensible estimates for the critical point $f_{c}$ with the approximations $P_{3,4}$, $P_{4,3}$. The lower estimate, 0.58999, is produced by $P_{4,3}$, while the slightly higher estimate, 0.59001, is found from $P_{3,4}$.

In such a case, with the latter estimate for the threshold $f_{c}\approx 0.59001$, in order to find the approximations for the index, a familiar minimal sensitivity condition from [24] is employed:

(72) $$\frac{\partial B_{7,0}\left(s\right)}{\partial s}=0,\;s=s_{7,0},$$

It does not include the Borel transformation. The critical index *s* is again the principal quantity of interest, and the effective conductivity is found by solving Equation (72). The solution to (72) can be found as follows:

$$k=7,\;\;\;\;s=s_{7,0}=0.28916.$$

To find the approximations for the index, a minimal difference condition could be imposed; see [24]:

(73) $$B_{7,0}\left(s\right)-B_{6,0}\left(s\right)=0,$$

and $s=s_{7,0}=s_{6,0}$ for k=7. In such a case, condition (73) does not include the Borel transformation at all. Thus, Equation (73) depends on the critical index by itself and allows us to find it simultaneously. The solution to (73) can be found as follows:

$$k=7,\;\;\;\;s=s_{7,0}=0.49053.$$

By taking their average, we estimate s≈0.38984±0.10069.

## 7. Discussion and Conclusions

In this paper, we build on our previous development of *a*RVE (analytical RVE) techniques for evaluating the effective conductivity of randomly dispersed composites. The method combines homogenization-based boundary value formulations with efficient computation of structural sums that reflect the spatial arrangement of inclusions. Earlier results expressed the effective conductivity tensor components as polynomial functions of the inclusion volume fraction *f*, valid from the dilute limit up to the critical concentration $f_{c}$ at which inclusions come into contact. As the system approaches this dense state, percolation pathways can emerge, and for high-contrast materials the originally bounded polynomial behavior may become singular, a phenomenon first reported by J.B. Keller [69]. Later extensions by Berlyand and Kolpakov [77,80,81] generalized this behavior to random composites and established an asymptotic framework for pairwise inclusion interactions of various shapes selected in Section 3.1. These advances ultimately led to the Network Method within the Structural Approximation (SA) framework. To align SA with homogenization principles, we introduce a periodic boundary value formulation defined on the associated Delaunay graph, allowing the method to address macroscopically anisotropic conductive composites.

In the present review, we summarize the *a*RVE methodology based on structural sums and renormalization to develop a strategy for determining the critical parameters $f_{c}$ and *s* in random composites [5,24]. It is worth noting that, for deterministic composites with fixed inclusion locations, the parameter $f_{c}$ can be determined relatively easily. In contrast, the problem becomes significantly more complex for random structures, where inclusion positions constitute a set of probabilistic events in both experimental observations and numerical simulations. A collection of real microstructural images may be treated as a statistical ensemble of realizations, while simulated structures are generated according to prescribed protocols that can yield varying estimates of $f_{c}$. In the present paper, particular emphasis is placed on elliptic inclusions with different statistical distributions. The resulting formulations are further refined using renormalization techniques to account for densely packed systems. Finally, the approach is generalized to polydisperse composites containing circular inclusions of varying sizes.

The critical index *s* can be used as a diagnostic tool to determine the degree and type of disorder imposed on the assemblages of monodispersed superconducting disks [24]. It turns out that for polydisperse composites, the index satisfies $0\ll s<\frac{1}{2}$, indicating that inclusion configurations are permeated with randomly positioned vacancies occupying the positions of inclusions at regular lattice sites [24].

The latter observation contrasts with the two cases involving elliptic inclusions, where *s* is closer to values reported in weakly disordered systems. Such values may be consistent with the ghost-lattice scenario discussed in [24]; however, the present results alone do not establish a unique correspondence between the numerical value of *s* and a specific microstructural class.

The critical exponent *s* should be viewed primarily as a quantitative descriptor of transport behavior near the critical threshold. Any connection between *s* and specific microstructural characteristics should be regarded as a working hypothesis supported by existing observations rather than as a universally established classification scheme.

While it is well-established that the critical exponent *s* depends fundamentally on the local microstructure, we further propose that the sample-to-sample variance in *s* serves as a quantitative diagnostic metric for structural disorder. In perfectly periodic lattices or idealized continuum approximations, *s* assumes a universal, predictable value. However, in densely packed random dispersions, localized fluctuations—specifically, the stochastic formation of narrow inter-particle gaps and localized clustering—induce measurable deviations from universality. We mathematically diagnose this disorder by evaluating the variance, Δs, across macroscopically identical realizations. As demonstrated quantitatively in our analysis of Variants A and B, maintaining a fixed concentration (f=0.38) and identical particle size distributions still yields a measurable divergence in the estimated exponent (Δs≈0.0185, with $s_{A}\approx 0.388$ and $s_{B}\approx 0.407$). Therefore, measuring the magnitude of this deviation provides a direct, quantifiable gauge of the hidden microscopic variance that is entirely missed by macroscopic descriptors such as total volume fraction. In this context, *s* does not map the exact geometric layout, but its departure from mean-field predictions mathematically diagnoses the severity of localized structural disorder governing the transport pathways.

In addition, we considered various DLog methods for estimating critical indices; see, e.g., [5,109,110]. In all three cases considered above, they yield results for the index *s* in the range between 0.3 and 0.54. The lower estimate follows from the standard method of [110], when both threshold and critical index ought to be found. The higher estimates follow from the methods advanced in [5], predicated on pre-knowledge of the threshold. The estimates of s≈0.54 hold for various approximants, Padé or iterated roots, for the threshold estimated from the Padé approximants as described above. Such direct estimates of the index *s* are in good agreement with the results of calculations presented above, based on consideration of the critical indices as control parameters to be determined from the minimal sensitivity and minimal-difference equations.

A continuous analytical model reveals general laws and functional dependencies rather than isolated numerical results, while substantially reducing the number of governing variables. Furthermore, the continuous averaging approach serves as a foundation for the development of discrete models, such as the two-particle interaction model [69,80,81] discussed in Section 3.1 and the Network Model presented in Section 3.2. The case of elliptical inclusions was first analyzed within the continuous framework, which subsequently led to a discrete description based on the singular behavior characterized by critical concentrations and the parameter *s*. Likewise, various physical models of media were treated using the same asymptotic renormalization methodology [114,115,119]. Therefore, continuous and discrete models should be viewed as complementary to provide analytical insight and asymptotic formulas, and computationally efficient and fast algorithms. Traditional numerical techniques, such as FEM, can then be employed to evaluate local fields in densely packed composites after the dominant singular behavior has been identified and extracted analytically.

As demonstrated throughout this review, the Structural Approximation and Network Model approaches are well developed for inclusions in $R^{d}$, primarily in the context of linear equations and smooth particle geometries. However, Kolpakov’s cone-plane pair discussed in Section 3.1 illustrates that gradient singularities may disappear when the inclusions have non-smooth boundaries. A similar phenomenon has been observed for the *p*-Laplacian. It would therefore be of considerable interest to investigate whether this effect persists for other classes of linear and nonlinear equations, as well as for more general types of non-smooth surfaces.

Most derivations within the *a*RVE framework have been carried out for 2D periodic cells with smooth particle boundaries. The theory has been successfully applied to 2D elasticity and 3D conductivity problems across the full range of contrast parameters. Furthermore, several experimentally reconstructed and observed microstructures were analyzed within the *a*RVE framework in [2,120], demonstrating its applicability to realistic heterogeneous materials.

To better understand the applicability and limitations of discrete and continuous models, consider the implications of the Banach–Tarski paradox, as discussed in [121] (p. 22). The paradox states that a solid ball can be partitioned into a finite number of disjoint subsets and subsequently reassembled into two balls without holes identical to the original one. This counterintuitive result relies on the existence of non-measurable sets, which cannot be assigned a meaningful volume and, consequently, cannot be associated with a physical mass. Such constructions are possible only within an idealized continuous mathematical framework. In contrast, physical objects possess an underlying discrete structure. For example, when a brittle stone fractures, it produces fragments with well-defined masses rather than arbitrary massless pieces. From this perspective, the Banach-Tarski paradox may be viewed as providing a conceptual justification for the discrete nature of an apparently continuous physical reality.

## Data Availability

The original contributions presented in this study are included in the article. Further inquiries can be directed to the corresponding authors.

## Abbreviations

The following abbreviations are used in this manuscript:

FEM	Finite Element Methods
AAM	Asymptotic Analytical Methods
*a*RVE	*analytical* Representative Volume Element
RSA	Random Sequential Addition
